# Cuticular Waxes in Altered Environments and Their Role in Leaf Water Loss Dynamics

**DOI:** 10.1111/pce.70714

**Published:** 2026-07-06

**Authors:** Lena Herzig, Lukas Schreiber, Paul Grünhofer

**Affiliations:** ^1^ Department of Ecophysiology, Institute of Cellular and Molecular Botany University of Bonn Bonn Germany

**Keywords:** abiotic stimuli, cuticle, cuticular transpiration, cuticular wax, environment, leaves, mutation, residual transpiration

## Abstract

Cuticular waxes undeniably form the primary barrier restricting uncontrolled water loss from leaves, yet the functional consequences of environmentally induced wax modifications for residual (cuticular) transpiration remain a matter of debate. This review summarises current knowledge on how major abiotic stimuli influence foliar cuticular wax amount and composition and critically re‐evaluates their effect on transpirational properties of leaves. By focusing on a strictly curated set of studies integrating gas chromatography for chemical wax analysis and permeance‐based residual (cuticular) transpiration measurements, we demonstrate that light quantity seems to represent the most dominant driver of wax deposition and that naturally increased wax amounts do not inherently translate into an enhanced water barrier, with the latter aspect challenging the recurrently postulated ‘more cuticular wax must equal lower rates of residual (cuticular) transpiration’ narrative. Instead, bridging modern research with long‐standing transport models indicates that the qualitative composition and spatial arrangement of cuticular wax might constitute the more decisive factors orchestrating cuticular water barrier properties. While the available data are still insufficient to identify clear causal relationships between compound classes, chain length distributions and structural heterogeneity affecting water permeability, they might point to further complexities, such as unintended off‐target effects as a consequence of genetic engineering.

## Summary statement

This review critically re‐evaluates how environmentally induced changes in foliar cuticular wax affect residual (cuticular) transpiration using a strictly curated dataset based on gas chromatographic wax analysis and permeance measurements. The compiled data show that increased wax amounts (despite being strongly stimulated by light and drought) do not inherently enhance the cuticular water barrier, but that the ultrastructure of the cuticle appears to play a more decisive role in determining its water permeability.

## 1 Introduction

The expansion of plants from aquatic to terrestrial habitats represents one of the most profound evolutionary shifts in the history of life on Earth. Amongst other factors, thriving on land required plants to balance two competing physiological needs: efficient water uptake from the soil and a strict control of water loss to the atmosphere. Water movement within and around plants is governed by differences in water potential, a measure of the free energy of water that determines the direction of its flow (Taiz and Zeiger 2003; Nobel 2020). If unhindered, water moves from compartments of higher to lower water potential, following a gradient that in terrestrial environments extends from the soil through the plant to the atmosphere. Under well‐watered conditions, the soil water potential is close to 0 MPa, while plant tissues typically maintain a lower water potential between −0.5 (e.g., roots) and −2 MPa (e.g., leaves), sufficient to passively draw water from irrigated substrates (Grünhofer and Schreiber 2023). In contrast, the surrounding air can reach values well below −100 MPa, creating an extreme gradient that maintains a continuous upward movement of water and nutrients through the plant body but simultaneously enforces a persistent evaporative demand from aerial tissues. Leaves are particularly affected, given that they constitute most of the plant's surface exposed to the dehydrating atmosphere (Whittaker and Likens 1975). At the same time, they must maintain gas exchange in order to perform photosynthesis, which inevitably increases the risk of water loss. In response, plants developed an evolutionarily peculiar system: all primary, atmosphere‐exposed organs are covered by the cuticle, a specialised hydrophobic layer that restricts uncontrolled water evaporation from the underlying tissue. Cuticles with structures and chemical compositions similar to those of many domesticated crop species have already been described in early‐diverging plant lineages such as bryophytes (Buda et al. 2013; Budke et al. 2011) and pteridophytes (Guo et al. 2018), suggesting that the cuticle is a highly conserved feature across embryophytes. In leaves of most vascular plants (with the exception of bryophytes, Merced and Renzaglia 2017), this protective membrane is perforated with stomatal pores that can be opened or closed in a highly dynamic manner and thus allow for a tightly regulated gas exchange (Kerstiens 1996). Even when stomata are fully closed, leaves continue to lose water at much lower but still measurable rates, a flux referred to as ‘minimum conductance’ or ‘residual transpiration’ (Schuster et al. 2017). This parameter represents the sum of all water loss pathways that persist under conditions of (maximum) stomatal closure, including leakage through imperfectly sealed stomata and diffusion across the cuticle. The latter component, termed ‘cuticular transpiration’ (liquid‐based permeance of approx. 5.0 *×* 10^−10^ m s^−1^), is often around two orders of magnitude lower than stomatal transpiration (liquid‐based permeance of approx. 9.5 *×* 10^−8^ m s^−1^) and defines the minimum quantity of water evaporation once stomata are fully closed during drought, darkness, or further stress conditions (Nobel 2020; Grünhofer and Schreiber 2023). Because residual transpiration was shown to be very comparable to pure cuticular transpiration in many species (Kerstiens 1996; Burghardt 2003; Burghardt et al. 2008; Grünhofer et al. 2022), but a minor contribution of still partially open stomata can hardly be excluded entirely, the term ‘residual (cuticular) transpiration’ will be used in this review.

The cuticle itself is a composite structure consisting of a cutin polymer matrix impregnated with hydrophobic cuticular waxes. It has been shown repeatedly that extraction of the soluble cuticular wax fraction using organic solvents (leaving behind only the insoluble cutin matrix) can increase the cuticular transpiration by two to three orders of magnitude, effectively shifting cuticular permeance into the same order of magnitude as stomatal transpiration (liquid‐based permeance of approx. 9.5 *×* 10^−8^ m s^−1^; Schönherr and Lendzian 1981; Schönherr and Riederer 1989; Grünhofer and Schreiber 2023). Cuticular waxes can be embedded into the cutin matrix (intracuticular) and deposited on top of the cuticle (epicuticular). In terms of their composition, intra‐ and epicuticular waxes are mainly composed of aliphatic lipids, while the intracuticular portion can also contain considerable amounts of pentacyclic triterpenoids (Zeisler‐Diehl et al. 2018). Even though cyclic compounds contribute to various protective functions, including UV shielding, pathogen defence and structural reinforcement of the cuticle, the current literature suggests that they are not the primary determinants of cuticular water permeability (Jetter and Riederer 2016; Seufert et al. 2021). Thus, this review will focus on the aliphatic fraction of cuticular wax.

The biosynthesis and assembly of the aliphatic cuticular wax components have been extensively investigated and are now, to a great extent, well understood at the genetic level (reviewed in, e.g., Kunst 2003; Kunst and Samuels 2009; Samuels et al. 2008; Yeats and Rose 2013; Lewandowska et al. 2020; Zhang et al. 2024) but will only be summarised briefly here. The biosynthesis of aliphatic cuticular wax takes place in the epidermal cells and starts with the plastidial de novo fatty acid synthesis, producing saturated C16 and C18 fatty acids that can be elongated by the microsomal fatty acid elongation machinery. During each consecutive elongation cycle, a C2 unit is added to the growing substrate, progressively extending the acyl chain by two carbon atoms to a maximum chain length of up to C34. These very‐long‐chain fatty acids can either be directly exported out of the cell or be further modified to yield monomeric primary and secondary alcohols, alkanes, aldehydes, ketones or dimeric esters. The final products are then exported and deposited into (intracuticular) or onto (epicuticular) the cutin matrix. Upon deposition, these compounds integrate into a structurally heterogeneous layer that determines the cuticle's physicochemical properties, including its water permeability and surface topography.

The multi‐step process of cuticular wax production is not static but responds to environmental cues. Despite being a non‐living interface, the cuticle exhibits a certain degree of plasticity that allows it to adapt to changes in the plant's abiotic environment. As sessile organisms, plants rely on such physiological flexibility to cope with environmental stressors. Climatic parameters such as light intensity, temperature and relative humidity vary not only across habitats but also seasonally and diurnally at a single site (Poorter et al. 2016). Ongoing (anthropogenic) climate change is expected to further amplify these fluctuations and the frequency of extreme events (IPCC 2023). This development will have a direct impact on plant–water relations, making it increasingly relevant to understand how cuticular wax is modulated by the environment and how this may affect cuticular water retention. Leaves will be focused on in this review, because they represent the primary site of transpirational water loss and are especially prone to influences from the environment.

Extensive previous research has demonstrated that environmental stimuli can significantly influence the amount and composition of foliar cuticular wax. However, a lack of consensus remains especially about the functional implications of such modifications for the transpirational barrier properties. In several past studies, enhanced wax accumulation was interpreted as an adaptive mechanism to mitigate water loss under stressful environmental conditions, but growing evidence suggests that this relationship is far more complex than previously assumed. Part of the challenge in reconciling these contradicting findings arises from methodological diversity among studies that assessed cuticular wax characteristics and their effect on residual (cuticular) transpiration. Differences in experimental design, (chemical) analytical approaches or data normalisation have often made it difficult to directly compare results across independent reports. To ensure that subsequent interpretations rest on a consistent and reliable foundation, this review will focus on a carefully curated dataset that meets pre‐defined quality standards.

## 2 Literature Selection

Considering the abundance of research investigating the relationships between environmental conditions and/or cuticular waxes and/or residual (cuticular) transpiration (109 publications were analysed), strict inclusion criteria for available publications had to be defined in order to enable and standardise comparisons between formerly independent studies. In this context, it should briefly be noted that the majority of selected data are based on highly bred model species, which have been genetically altered during domestication or even by targeted genetic modifications, and thus cannot entirely capture the existing biological and ecological diversity. Nonetheless, the filtered studies still cover a diverse range of ecologically and economically important species that provide a robust foundation for our reassessment. Apart from a generally hypothesis‐driven study layout (effect of environment **→** wax vs. effect of wax **→** transpiration), great emphasis was placed on the utilisation of state‐of‐the‐art methodologies of chemical analysis and/or residual (cuticular) transpiration measurements. The inclusion criteria (refer to the Supporting Information Section S2) comprised (i) restriction of interspecific variation (Subsection S2.1), (ii) emphasis on whole leaves (Subsection S2.2), (iii) application of gas chromatography for high‐quality cuticular wax analysis, and also accounting for potential differences in leaf surface area (Subsection S2.3), and (iv) permeance estimation as a robust and standardised metric for quantifying residual (cuticular) transpiration (Subsection S2.4). All studies that were screened considering the established inclusion criteria and ultimately either included (31 publications, incorporated into Tables 1, 2, 3) or rejected (78 publications) are listed in Table S1.

**Table 1:** Effect of major environmental stimuli on the surface‐area‐normalised absolute cuticular wax amount and the relative cuticular wax composition of leaves. To ensure comparability across the compiled data and provide high‐quality results, only research that employed state‐of‐the‐art analytical methods was included. Studies based on gravimetric methods are separately compiled in Table S2. Approximate values were estimated from figures when no numerical data were available. Parentheses […] were added in cases where statistical significance was reported in the original study, but the relevance of the response is questioned by the authors of this review: significant changes in the absolute wax amount were categorised as ‘meaningful’ at a threshold of > 1.25‐fold or < 0.75‐fold (± 25% of the control treatment wax amount), and significant changes in the relative wax composition were categorised as ‘meaningful’ when the relative abundance of at least one compound class changed by more than ±10 percentage points. If several cultivars of the same species were tested in parallel within one study (indicated by cvv.), the response was categorised as ‘meaningful’ if it was observed consistently in the majority of cultivars.

Species	Leaf state	Stimulus	Details	Effect on wax amount	Absolute change	Effect on wax composition	Relative change	Reference
*Brassica oleracea*	FD	Light exposure	38 vs. 80 W m^−2^	↑	2.30‐fold	No	No change	Baker (1974)
*Hordeum vulgare*	FD	Light exposure	100 vs. 800 µmol m^−2^ s^−1^	↑	1.67‐fold^a^	No	No change	Herzig et al. (2025)
*Zea mays*	FD	Light exposure	100 vs. 1200 µmol m^−2^ s^−1^	↑	2.30‐fold^a^	No	No change	Herzig et al. (2025)
*Solanum lycopersicum*	FD	Light exposure	50 vs. 800 µmol m^−2^ s^−1^	↑	2.44‐fold^a^	No	No change	Herzig et al. (2025)
*Vigna radiata*	FD	Light exposure	100 vs. 800 µmol m^−2^ s^−1^	↑	4.64‐fold^a^	No	No change	Herzig et al. (2025)
*Ulmus pumila*	FD	Light exposure	Shade‐ vs. sun‐leaves	↑	1.56‐fold	Yes	−10 pp acids +12 pp sum of alkanes and alcohols	Lykholat et al. (2020)
*Ulmus minor*	FD	Light exposure	Shade‐ vs. sun‐leaves	↑	2.63‐fold	Yes	−25 pp acids +20 pp sum of alkanes and alcohols	Lykholat et al. (2020)
*Arabidopsis thaliana*	NA	Light exposure	Darkness vs. light	[↑]	Approx. 1.2‐fold	No	No change	Go et al. (2014)
*Phaseolus vulgaris*	FD	UV‐B radiation	3.4 vs. 8 kJ m^−2^ d^−1^	↑	1.28‐fold	No	No change	Steinmüller and Tevini (1985)
*H. vulgare*	FD	UV‐B radiation	3.4 vs. 8 kJ m^−2^ d^−1^	[↑]	1.23‐fold	[Yes]	Approx. +2 pp aldehydes, −8 pp alcohols	Steinmüller and Tevini (1985)
*A. thaliana*	NA	Low temperature	22°C vs. 5°C	↓	0.62‐fold	Yes	Approx. +30 pp acids, −30 pp alkanes	Rahman et al. (2021)
*B. oleracea*	FD	Low temperature	21°C vs. 15°C	↓	0.74‐fold	Yes	+10 pp alkanes	Baker (1974)
*Thellungiella salsuginea*	NA	Low temperature	22°C vs. 4°C	[↑]	1.15‐fold	[Yes]	+6 pp acids, −4 pp alkanes	He et al. (2019)
*B. oleracea*	FD	High temperature	21°C vs. 35°C	[↓]	0.80‐fold	[Yes]	+7 pp aldehydes, −4 pp alkanes	Baker (1974)
*B. oleracea*	FD	Low relative humidity	70% vs. 40%	[↑]	1.10‐fold	No	No change	Baker (1974)
*B. oleracea*	E/FD	Low relative humidity	40%–70% vs. 20%–30%	[↑]	1.05‐fold	No	No change	Koch et al. (2006)
*Tropaeolum majus*	E/FD	Low relative humidity	40%–70% vs. 20%–30%	[↑]	1.23‐fold	No	No change	Koch et al. (2006)
*Eucalyptus gunnii*	E/FD	Low relative humidity	40%–70% vs. 20%–30%	[↑]	1.15‐fold	No	No change	Koch et al. (2006)
*B. oleracea*	FD	High relative humidity	70% vs. 98%	↓	0.48‐fold	[Yes]	+4 pp aldehydes, −6 pp ketones	Baker (1974)
*B. oleracea*	E/FD	High relative humidity	40%–70% vs. 98%	↓	0.55‐fold	Yes	Approx. +12 pp ketones	Koch et al. (2006)
*E. gunnii*	E/FD	High relative humidity	40%–70% vs. 98%	[↓]	0.98‐fold	No	No change	Koch et al. (2006)
*T. majus*	E/FD	High relative humidity	40%–70% vs. 98%	[↓]	0.93‐fold	No	No change	Koch et al. (2006)
*Nicotiana glauca*	FD	Simulated drought	Dehydration until visible wilting	↑	Approx. 2.5‐fold	No	No change	Cameron et al. (2006)
*Camellia sinensis*	E	Simulated drought	No watering for 15 days	↑	Tender: 1.94‐fold	Yes	Tender: +19 pp alkanes, −26 pp ols	Chen et al. (2020)
*C. sinensis*	FD	Simulated drought	No watering for 15 days	↑	Mature: 1.24‐fold	Yes	Mature: +14 pp alkanes	Chen et al. (2020)
*C. sinensis* (cvv.)	FD	Simulated drought	No watering for 12 days	↑	1.53‐ to 3.4‐fold across 3 cultivars	?	Separate analyses of adaxial, abaxial, intra‐ and epicuticular waxes	Zhang et al. (2021)
*Ammopiptanthus mongolicus*	NA	Simulated drought	Watering with 20% PEG solution for 14 days	↑	1.72‐fold	[Yes]	Approx. +3 pp alkanes	Sumbur et al. (2024)
*Gossypium hirsutum*	FD	Simulated drought	70% vs. 10% soil RWC	↑	1.58‐fold	Yes	+12 pp alkanes, −12 pp acids	Yang et al. (2022)
*Glycine max* (cvv.)	E/FD	Simulated drought	No watering for 5 days in two subsequent periods	↑	1.29‐fold based on means of 18 cultivars	No	No change based on means of 18 cultivars	Kim et al. (2007a)
*Sesamum indicum* (cvv.)	E/FD	Simulated drought	No watering for 15 days	↑	1.29‐fold based on means of 18 cultivars	No	No change based on means of 18 cultivars	Kim et al. (2007b)
*H. vulgare* (cvv.)	FD	Simulated drought	−0.04 vs. −0.8 MPa	↑	1.30‐ and 1.50‐fold in 2 cultivars	No	No change in 2 cultivars	Shellakkutti et al. (2022)
*A. thaliana*	E/FD	Simulated drought	No watering for 7–9 days	↑	1.75‐fold	No	No change	Kosma et al. (2009)
*A. thaliana*	E/FD	Simulated drought	No watering for 7 days	=	No change	=	No change	Patwari et al. (2019)
*Ricinus communis* (cvv.)	FD	Simulated drought	75% vs. 25% pot capacity	[↑]	1.22‐ and 1.23‐fold in 2 cultivars	[Yes]	+5 pp acids, −8 pp alcohols in 1 of 2 cultivars	de Araújo Silva et al. (2021)
*Rosa* × *hybrida* (cvv.)	FD	Simulated drought	Reduced watering for 10 days in three subsequent periods	[↑]	1.08‐ and 1.09‐fold in 2 cultivars	[Yes]	Approx. +3 pp acids in 1 of 2 cultivars	Jenks et al. (2001)
*Medicago sativa* (cvv.)	FD	Simulated drought	75%–85% vs. 25%–35% soil RWC	=	No change in 6 cultivars	Yes	Approx. +10 to +18 pp alkanes in 3 out of 6 cultivars, approx. −11 to −25 pp alcohols in 4 out of 6 cultivars	Ni et al. (2012)
*A. thaliana*	E/FD	NaCl exposure	0 vs. 150 mM	↑	1.80‐fold	No	No change	Kosma et al. (2009)
*T. salsuginea*	E/FD	NaCl exposure	0 vs. 300 mM	↑	4.08‐fold	No	No change	Zhang et al. (2022)
*T. salsuginea*	E/FD	NaCl exposure	0 vs. 150 mM	[↑]	1.08‐fold	No	No change	Zhang et al. (2022)
*H. vulgare*	FD	NaCl exposure	0 vs. 100 mM	=	No change	No	No change	Fricke et al. (2006)
*H. vulgare*	FD	NaCl exposure	0 vs. 150 mM	=	No change	No	No change	Even et al. (2018)
*H. vulgare* (cvv.)	FD	NaCl exposure	0 vs. 180 mM	=	No change in 2 cultivars	No	No change in 2 cultivars	Grünhofer et al. (2025)

Abbreviations: ?, the study reported compositional data but not in a form that allowed categorical assessment; E, expanding leaves; E/FD, mixture of expanding and fully developed leaves; FD, fully developed leaves; NA, not available; pp, percentage points; RWC, relative water content.

^a^ Comparing the minimum observed wax amounts (identified in light treatment of 50 or 100 µmol m^−2^ s^−1^) against the maximum observed wax amounts (identified in light treatment of 800 or 1200 µmol m^−2^ s^−1^) across 6 light treatments in total (50, 100, 200, 400, 600, 800, and 1200 µmol m^−2^ s^−1^).

**Table 2:** Effect of alterations in the surface‐area‐normalised absolute cuticular wax amount on the residual (cuticular) transpiration of intact and fully developed leaves. To ensure comparability across the compiled data and provide high‐quality results, only research that employed state‐of‐the‐art analytical methods in combination with permeance measurements was included. Parentheses […] were added in cases where statistical significance was reported in the original study, but the relevance of the response is questioned by the authors of this review: significant changes in the absolute wax amount were categorised as ‘meaningful’ at a threshold of > 1.25‐fold or < 0.75‐fold (±25% of the control treatment wax amount), and significant changes in permeance were categorised as ‘meaningful’ at a threshold of > 1.25‐fold or < 0.75‐fold (±25% of the control treatment permeance).

Species	Mutation or stimulus	Details	Change in wax amount	Absolute change	Change in wax composition	Effect on water permeance	Absolute change	Control of stomatal contribution	Reference
*Populus* × *canescens* (cv. 84K)	Cultivation conditions	Early climate chamber vs. outdoor cultivation	↑	12.69‐fold#	No	=	No change	Comparison of WLs to CMs	Grünhofer et al. (2022)
*Quercus coccifera*	Climatic regions	Mediterranean vs. temperate climate	↑	2.68‐fold	No	=	No change	Plateau selection from WL kinetics	Bueno et al. (2019a)
*V. radiata*	Light exposure	100 vs. 800 µmol m^−2^ s^−1^	↑	4.64‐fold+	No	=	No change	Plateau selection from WL kinetics	Herzig et al. (2025)
*S. lycopersicum*	Light exposure	50 vs. 800 µmol m^−2^ s^−1^	↑	2.44‐fold+	No	↑	3.20‐fold	Plateau selection from WL kinetics	Herzig et al. (2025)
*Z. mays*	Light exposure	100 vs. 1200 µmol m^−2^ s^−1^	↑	2.30‐fold+	No	=	No change	Plateau selection from WL kinetics	Herzig et al. (2025)
*H. vulgare*	Light exposure	100 vs. 800 µmol m^−2^ s^−1^	↑	1.67‐fold+	No	=	No change	Plateau selection from WL kinetics	Herzig et al. (2025)
*H. vulgare* (cvv.)	Simulated drought	−0.04 vs. −0.8 MPa	[↑]	1.20 and 1.31‐fold in 2 cultivars	No	=	No change in 2 cultivars	Plateau selection from WL kinetics	Shellakkutti et al. (2022)
*Populus trichocarpa*	Seasonality	May vs. September	↓	0.44‐fold	Yes*	↓	0.57‐fold	Plateau selection from WL kinetics	Grünhofer et al. (2021)
*Populus euphratica*	Seasonality	May vs. September	↓	0.52‐fold	No	↓	0.56‐fold	Plateau selection from WL kinetics	Grünhofer et al. (2021)
*Populus* × *canescens* (cvv.)	Seasonality	May vs. September	↓	0.75‐ and 0.41‐fold in 2 cultivars	No	[↓]	0.48‐fold in 1 out of 2 cultivars	Plateau selection from WL kinetics	Grünhofer et al. (2021)
*Dianthus caryophyllus* (cv. Master)	Ontogenetic development	Flowering stage I vs. II	↓	0.53‐fold	No	=	No change	Not specified	Cheng et al. (2023)
*H. vulgare*	Reductase‐knock‐out	*cer‐za.227* (*Hvfar1*)	↓	0.30‐fold	No	=	No change	Plateau selection from WL kinetics	Müller et al. (2023)
*H. vulgare*	Elongase‐knock‐out	*cer‐ye.267* (*Hvkcs1*)	↓	0.30‐fold	No	↑	1.44‐fold	Plateau selection from WL kinetics	Müller et al. (2023)

Abbreviations: CM, cuticular membrane; WL, whole leaf.

* Also affected in the relative wax composition, the respective data are shown in Table 3.

^#^ Comparing ex vitro climate chamber‐developed leaves to 6‐week‐old leaves developed outdoors.

^+^ Comparing the minimum observed wax amounts (identified in light treatment of 50 or 100 µmol m^−2^ s^−1^) against the maximum observed wax amounts (identified in light treatment of 800 or 1200 µmol m^−2^ s^−1^) across 6 light treatments in total (50, 100, 200, 400, 600, 800 and 1200 µmol m^−2^ s^−1^).

**Table 3:** Effect of alterations in the relative cuticular wax composition on the residual (cuticular) transpiration of intact and fully developed leaves. To ensure comparability across the compiled data and provide high‐quality results, only research that employed state‐of‐the‐art analytical methods in combination with permeance measurements was included. Parentheses […] were added in cases where statistical significance was reported in the original study, but the biological relevance of the response is questioned by the authors of this review: significant changes in the relative wax composition were categorised as ‘meaningful’ when the relative abundance of at least one compound class changed by more than ±10 percentage points, and significant changes in permeance were categorised as ‘meaningful’ at a threshold of > 1.25‐fold or < 0.75‐fold (±25% of the control treatment permeance).

Species	Mutation or stimulus	Details	Change in wax amount	Change in wax composition	Relative change	Effect on water permeance	Absolute change	Control of stomatal contribution	Reference
*Populus* × *canescens* (cv. INRA 717‐1B4)	Elongase‐knock‐out	*cer6*	=	Yes	> 90% esters, wMCL from 34.1 to 40.7	↑	1.45‐fold	Comparison of WLs to LDs	Grünhofer et al. (2023)
*D. caryophyllus* (cv. Lady green)	Ontogenetic development	Flowering stage I vs. II	=	[No]	+17 pp alcohols, no wMCL analysis	↑	1.93‐fold	Not specified	Cheng et al. (2023)
*P. trichocarpa*	Seasonality	May vs. September	↓*	[No]	+15 pp alkanes, no change in wMCL	↓	0.57‐fold	Plateau selection from WL kinetics	Grünhofer et al. (2021)

Abbreviations: LD, leaf disc; pp, percentage points; WL, whole leaf; wMCL, weighted mean chain length.

* Also affected in the absolute wax amount, the respective data are shown in Table 2.

Considering all factors outlined in the Supporting Information (Section S2), we have identified some key methodological shortcomings that need to be accounted for before attempting to newly evaluate how environmental stimuli influence the foliar cuticular wax amount and composition (Section 3) and how such changes in either the absolute cuticular wax amount (Section 4) or its relative composition (Section 5) relate to residual (cuticular) transpiration. By applying the inclusion criteria defined above, we aim to converge on what the ‘true relationship’ of cuticular wax and cuticular water permeability actually looks like and clearly distinguish it from observations that might rather reflect experimental artefacts. Ultimately, this review will aim to present hypotheses to help explain the observed relationships, or lack thereof (Section 6).

## 3 Cuticular Wax Is Subject to the Environment

As the outermost barrier of plants, the cuticle is directly exposed to—and shaped by—the surrounding environment. Comprehensive research has highlighted that the amount and composition of leaf cuticular wax are not static but can be modulated in response to altered cultivation conditions: For instance, Grünhofer et al. (2022) reported that poplars (*Populus × canescens*) cultivated outdoors under natural conditions accumulated 12.69‐fold more wax than those grown in the highly artificial setting of a climate chamber. Similarly, Sutter and Langhans (1982) found gravimetry‐based 6.44‐fold and 11.34‐fold more cuticular wax loads on leaves of cabbage (*Brassica oleracea*) seedlings from the greenhouse and outdoors, respectively, compared to seedlings cultivated in vitro. These exemplary findings illustrate that cuticular wax biosynthesis is strongly modulated by surrounding environments, which are characterised not only by a complex interplay of different abiotic factors, but also by pronounced seasonal and diurnal fluctuations in such factors—natural variability that is often minimised or absent and regularly ignored in artificial growth systems. While this environmental complexity is an integral component of natural ecosystems, it can be difficult for scientists to quantify or replicate it (Poorter et al. 2016). To address this problem, experimental research typically employs a reductionist approach, focusing on the effect of individually selected climatic factors in an otherwise controlled environment. While this approach substantially simplifies the complexity of natural environments and might therefore yield results that cannot necessarily be extended to field conditions (Plessis 2023), it nonetheless provides valuable insights into how specific stimuli shape the developmental processes in plants and may ultimately contribute to our understanding of plant–environment interactions. In the following subsections, we have summarised literature investigating the effect of major environmental variables on the absolute cuticular wax amount and its relative composition (Table 1) and aim to provide a synoptical overview of how exactly cuticular wax is influenced by the environment.

### 3.1 Light Exposure

Dating back the scientific interests to at least half a century ago, it is nowadays well‐documented that light is a key external driver of the cuticular wax deposition (Baker 1974; Lykholat et al. 2020; Herzig et al. 2025). While light quality can influence wax biosynthesis to some extent (Huang et al. 2020), light quantity appears to be the more significant determinant of the cuticular wax deposition, with accumulating evidence from various species (Table 1). In *B. oleracea*, for instance, Baker (1974) demonstrated that fully developed leaves exhibited a 2.30‐fold increase in the absolute wax amount when the light intensity was doubled (38 vs. 80 W m^−2^). More recently, a comprehensive study investigating four different species in parallel confirmed a clear positive correlation between light quantity and cuticular wax accumulation in *Hordeum vulgare*, *Zea mays*, *Solanum lycopersicum* and *Vigna radiata*, with responses ranging from 1.67‐fold (*H. vulgare*) up to 4.64‐fold (*V. radiata*) of increased wax loads (Herzig et al. 2025). Comparable trends were reported for two types of leaves on the same tree of the woody species *Ulmus pumila* and *Ulmus minor*, where sun‐exposed leaves accumulated 1.56‐fold (*U. pumila*) and 2.63‐fold (*U. minor*) more wax compared to shaded ones (light intensities were not further quantified; Lykholat et al. 2020). Cultivation of *Arabidopsis thaliana* in ‘darkness’ compared to ‘light’ (not further quantified; Go et al. 2014) resulted in a mild accumulation of cuticular wax by approximately 1.2‐fold. A positive correlation between light quantity and wax deposition is further supported by widely referenced gravimetric studies, which were excluded from this review due to their lower precision (Section 2) but can be reviewed separately in Table S2.

While the stimulatory effect of light quantity on the cuticular wax amount is consistent across different species, its influence on the relative wax composition appears to be more species‐specific. In *U. pumila* and *U. minor*, for example, the sum of alkanes and alcohols increased (between +12 and +20 percentage points) and the relative abundance of fatty acids decreased (between −10 and −25 pp) on sun‐exposed leaves (Lykholat et al. 2020). Contrastingly, increasing light quantity did not affect the cuticular wax composition in *B. oleracea*, *H. vulgare, Z. mays, S. lycopersicum* and *V. radiata* (Baker 1974; Herzig et al. 2025).

Collectively, the available data convincingly show that light quantity is a strong and uniform external driver of quantitative wax accumulation, while it does not necessarily induce considerable qualitative modifications of the relative wax composition in most investigated species.

### 3.2 UV‐B Radiation

Remarkably, the currently reported effect of UV‐B radiation on wax biosynthesis is not as prominent. Although the cuticular wax layer contributes to UV‐B protection (Mulroy 1979), UV‐B radiation itself does not appear to directly stimulate the deposition of cuticular wax (Table 1). Steinmüller and Tevini (1985) demonstrated that a selective increase in UV‐B exposure (in the spectrum between 280 and 360 nm) induced only a mild increase in cuticular wax loads in *H. vulgare* (1.23‐fold) and *Phaseolus vulgaris* (1.28‐fold), while it had little to no effect on the relative cuticular wax composition. Apart from this study, chemical analytical investigations examining the cuticular wax in response to UV‐B are scarce. Additional gravimetric data from Table S2 indicate that the effect of UV‐B on the cuticular wax amount is inconsistent across different species and even cultivars of the same species.

The combined collection of available data did not reveal any clear and consistent effect of UV‐B radiation on the cuticular wax biosynthesis. It appears that plants react stronger to overall light quantity in the visible spectrum (Subsection 3.1) than to UV‐B radiation in isolation, but the underlying factors require further investigation.

### 3.3 Temperature

Temperature is a fundamental environmental parameter that shapes plant development, yet its role in regulating cuticular wax deposition remains less well understood compared to that of light. Previous reviews have reported that cold conditions often enhance cuticular wax biosynthesis (Shepherd and Wynne Griffiths 2006; Shaheenuzzamn et al. 2021); however, this conclusion was largely based on either gravimetric data (which lacks appropriate qualitative resolution) or refers to comparatively mild increases in wax amounts (below 1.25‐fold). Instead, a collection of studies conforming to the inclusion criteria defined in Section 2 (Table 1) reported an opposite trend: In *A. thaliana*, for instance, a decrease in temperature from 22°C to 5°C reduced the wax load on rosette leaves to 0.62‐fold (Rahman et al. 2021). A similar response was observed in *B. oleracea* (reduction to 0.74‐fold; Baker 1974) when temperatures were decreased from 21/23°C to 15°C. He et al. (2019) observed increased cuticular wax deposits on *Thellungiella salsuginea* leaves when the plants were cultivated under cold conditions (22°C vs. 4°C); however, the amounts only increased by 1.15‐fold. Nonetheless, it deserves attention that a decrease in temperature does not necessarily result in decreased wax loads in all species, but that cold sensitivity appears to be species‐specific and not at all uniform (Tables 1 and S2). In response to elevated temperatures, equally inconsistent effects were reported, even though less literature is available on that topic (Tables 1 and S2).

Although temperature does not seem to be a strong regulator of the absolute wax amount, it appears to affect the relative composition to a greater extent. In *A. thaliana*, the relative proportion of fatty acids increased under cold stress (22°C vs. 5°C, approximately +30 pp), while the abundance of alkanes decreased (approximately −30 pp; Rahman et al. 2021). A similar trend, although much less pronounced, was observed in *T. salsuginea* (22°C vs. 4°C, +6 pp fatty acids, −4 pp alkanes; He et al. 2019). In *B. oleracea*, in contrast, the abundance of alkanes increased at moderately low temperatures (21°C vs. 15°C, +10 pp alkanes), while only mild shifts in wax composition were observed in response to high temperatures (21°C vs. 35°C, +7 pp aldehydes, −4 pp alkanes; Baker 1974).

Despite an overall more obvious response in wax quality than quantity, the partly conflicting results reported for different species indicate a high degree of species dependence. Considering the limited number of chemical analytical studies that address this topic, further research is needed to unravel the mechanisms behind temperature‐induced changes in cuticular wax biosynthesis. In comparison, more is known about the impact of temperature on the three‐dimensional structure of epicuticular wax crystals and thus the foliar surface topography (Whitecross and Armstrong 1972; Reed and Tukey 1982; Gauvrit and Gaillardon 1991). Beyond affecting wax biosynthesis, temperature therefore likely represents an important additional determinant in modulating foliar surface properties such as wettability and pathogen or herbicide retention (Koch and Barthlott 2006; Chiu et al. 2020).

### 3.4 Relative Humidity

The concentration of atmospheric water vapour plays a significant role in a plant's water status because it determines how strongly water is drained from the above‐ground tissues, especially when stomata are open. Young and fragile plantlets, which often lack the ability to regulate stomatal closure (Conner and Conner 1984; Santamaria and Kerstiens 1994), are thus commonly cultivated by using in vitro tissue culture or under transparent covers at humidities close to 100% and need to be gradually acclimatised to decreasing relative humidities. It has been shown before that acclimatisation of in vitro plantlets to climate chamber conditions can coincide with a significant increase in the cuticular wax coverage, especially of newly developing ex vitro leaves (Kerstiens 1994; Sutter and Langhans 1982; Grünhofer et al. 2022). However, this reaction cannot be conclusively assigned to changes in relative humidity alone, given that plantlets growing in tissue culture are still in an early ontogenetic stage during which cuticle maturation might still be ongoing and increasing wax loads could simply reflect developmental progression. To avoid confounding developmental effects, experiments investigating changes in relative humidity should ideally be conducted with fully acclimatised ex vitro plants. When screening the available literature for studies that fulfill these requirements, the resulting dataset (Table 1) demonstrates that a selective decrease in relative humidity (here always compared to control conditions between 40% and 70%) can induce a mild accumulation of cuticular waxes, as it was shown for *B. oleracea* (1.10‐fold and 1.05‐fold; Baker 1974; Koch et al. 2006), *Eucalyptus gunnii* (1.15‐fold; Koch et al. 2006) and *Tropaeolum majus* (1.23‐fold; Koch et al. 2006). Conversely, an increase in relative humidity to 98% considerably decreased cuticular wax loads in *B. oleracea* to 0.48‐fold and 0.55‐fold of the original amount, while the wax loads of *E. gunnii* (0.98‐fold) and *T. majus* (0.93‐fold) remained nearly unchanged (Baker 1974; Koch et al. 2006).

The relative wax composition was not affected by relative humidity in any case except for *B. oleracea*, which exhibited an increase in ketones (approximately +12 pp ketones) when cultivated at 98% relative humidity compared to 40%–70% control conditions (Koch et al. 2006). However, the study by Baker (1974) that applied a similar treatment (70 vs. 98% relative humidity) and investigated the same species did not confirm this qualitative shift but even reported a mild trend in the opposite direction (−6 pp ketones, +4 pp aldehydes). Thus, it remains questionable to what extent this observation in *B. oleracea* actually constitutes a reproducible response.

The compiled data indicate that low relative humidity has a mild stimulatory effect on the accumulation of cuticular wax, while high relative humidity appears to favour low cuticular wax deposits, especially in *B. oleracea*. Nonetheless, the specific responses are nuanced, and the functional significance remains questionable. The cuticular wax composition was largely unaffected by changes in relative humidity.

### 3.5 Simulated Drought

While the atmospheric humidity determines water evaporation from above‐ground organs, the rhizospheric water availability dictates the capacity to not only equally compensate for this loss, but also provide surplus quantities to support biological processes. The uptake by roots and the loss by shoots need to be perfectly balanced in order to maintain the plant's water status and ensure prolonged plant survival, and even a slight disruption of that balance can lead to water deficits, causing severe stress (Taiz and Zeiger 2003). In drought stress experiments, providing optimum comparability is especially challenging because there is no uniformly accepted definition of what ‘drought stress’ actually means and how respective treatments should be designed. Thus, in this review, we had to compare different kinds of experimental approaches trying to simulate natural droughts. For example, many studies in this field of research apply a ‘strategic’ water withdrawal procedure without exactly quantifying its intensity by means of water potential measurements, which certainly needs to be considered when the results are interpreted and compared. Nonetheless, the acquired data still appear to be sufficiently indicative to reveal general trends in the regulation of cuticular wax biosynthesis.

The previous subsection emphasised that cuticular wax is not quite responsive to low atmospheric humidities. A decreased rhizospheric water supply, however, appears to have a more pronounced effect on the cuticular wax accumulation, although some contradiction still persists (Table 1): In *Nicotiana glauca*, several dehydration events enhanced wax deposition by approximately 2.5‐fold (Cameron et al. 2006). Similarly, withholding irrigation resulted in 1.24‐ to 3.40‐fold accumulated cuticular wax amounts in *Camellia sinensis* (across leaf ages and cultivars; Chen et al. 2020; Zhang et al. 2021). In *Ammopiptanthus mongolicus*, exerting osmotic stress by watering plants with polyethylene glycol solution increased wax deposition by 1.72‐fold (Sumbur et al. 2024). In *Gossypium hirsutum*, a decrease in the soil relative water content from 70% to 10% led to increased wax loads by 1.58‐fold (Yang et al. 2022). Across 18 cultivars of *Glycine max* (Kim et al. 2007a), withholding watering elevated the mean wax loads by 1.29‐fold, and the same reaction was observed across 18 cultivars of *Sesamum indicum* (Kim et al. 2007b). In *H. vulgare*, decreasing the osmotic potential in hydroponics from −0.04 to −0.8 MPa increased the cuticular wax deposits between 1.30‐fold and 1.50‐fold in two cultivars (Shellakkutti et al. 2022). Interestingly, Shellakkutti et al. (2022) suggested that increased wax amounts in response to osmotic stress may partly result from reduced leaf expansion, effectively distributing the same total wax over a smaller leaf surface area. Simultaneously, molecular analyses from the same study revealed that the expression of wax‐related genes is indeed responsive to osmotic stress, which coincides with other recent findings (Sumbur et al. 2024). It appears that increasing wax amounts per unit surface area, specifically following osmotic stress, likely results from a combination of enhanced wax production and decreased leaf surface area. Although these copious findings indicate a uniform drought response in the cuticular wax production across various species, some exceptions have been reported: In *A. thaliana*, for example, withholding irrigation increased cuticular wax amounts by 1.75‐fold in one study (Kosma et al. 2009), while the same treatment did not affect cuticular wax amounts in another (Patwari et al. 2019). Overall, milder effects of simulated drought on the cuticular wax deposition were observed in *Ricinus communis* (1.22‐fold and 1.23‐fold; de Araújo Silva et al. 2021) and *Rosa* × *hybrida* (1.08‐fold and 1.09‐fold; Jenks et al. 2001). *Medicago sativa* was the only species where wax loads were not affected by drought conditions following a decreased soil relative water content from approximately 80% to 30% (Ni et al. 2012).

An effect on the relative wax composition was only observed in three of the investigated species, namely *C. sinensis, G. hirsutum* and *M. sativa*. All three showed an increase in alkanes in response to simulated drought; however, *G. hirsutum* additionally exhibited decreased amounts of fatty acids (+12 pp alkanes, −12 pp fatty acids in *G. hirsutum*; Yang et al. 2022), while *C. sinensis* and *M. sativa* showed a reduced fraction of primary alcohols (+19 pp alkanes, −26 pp alcohols in tender leaves, +14 pp alkanes in mature leaves of *C. sinensis*; Chen et al. 2020; approximately +18 pp alkanes, approximately −25 pp alcohols in *M. sativa*; Ni et al. 2012). In all other investigated species, the relative wax composition was not affected to a meaningful degree.

While defining and standardising drought stress remains challenging, the experimental approaches reviewed here demonstrate that reduced rhizospheric water availability can trigger an accumulation of cuticular wax in a range of different species, although the magnitude of this response is variable. The wax composition was only affected in three of the investigated species, indicating that the primary drought‐induced response lies in quantitative rather than qualitative changes.

### 3.6 NaCl Exposure

Increasing salinity by NaCl exposure exerts an even more intricate challenge for plants, thus inducing a range of complex physiological and biochemical responses. Among these, alterations in the cuticular wax have been reported for *A. thaliana* and *T. salsuginea* (Table 1). In *A. thaliana*, treatment with 150 mM NaCl in hydroponic culture resulted in 1.80‐fold increased wax amounts on rosette leaves (Kosma et al. 2009). In the halophyte *T. salsuginea*, irrigating plants on soil with nutrient solution containing 150 mM NaCl did not induce a notable reaction, but when the supplemented salt concentration was increased to 300 mM, *T. salsuginea* plants exhibited a pronounced 4.08‐fold increase in cuticular wax amounts (Zhang et al. 2022). However, in *H. vulgare*, exposure to 100, 150 or 180 mM NaCl in hydroponics performed throughout three independent studies did not induce a significant increase in the cuticular wax amount in any case (Fricke et al. 2006; Even et al. 2018; Grünhofer et al. 2025).

None of the mentioned studies found changes in wax composition in response to NaCl exposure. Although the available collective data are limited, they currently indicate that NaCl treatment can induce an acceleration of the cuticular wax deposition in some species, but without altering its composition.

Interestingly, despite the fact that NaCl exposure (combining osmotic stress with ion toxicity) exerts even broader physiological hardship than osmotic stress alone, the reactions in cuticular wax were comparatively weak. It is possible that plants might allocate resources to internal ion homoeostasis rather than strengthening the external cuticular barrier under salt stress conditions, but the specific response appears to depend on the species and their respective evolutionary adaptation to high salt concentrations.

### 3.7 Multifactorial Environments

The previous discussions have shown that changing individual abiotic factors in an otherwise tightly controlled environment can influence cuticular wax quantity and quality across different species (Tables 1 and S2). However, the artificial experimental designs in climate chambers with constant irradiation, humidity, airflow and temperature regimes differ profoundly from realistic outdoor environments, where plants are continuously exposed to highly dynamic and multifactorial abiotic influences. For instance, increasing irradiation (Section 3.1) usually also coincides with elevated UV‐B exposure (Section 3.2), higher temperatures (Section 3.3), stronger vapour pressure deficit (Section 3.4) and sometimes also reduced rhizospheric water availability (Section 3.5). Consequently, the cuticular wax phenotype observed under natural conditions likely always reflects the integrated outcome of numerous interacting abiotic influences rather than the response to a single factor (Mittler 2006).

While studies applying a reductionistic approach are abundant, investigations under natural environmental conditions remain comparatively scarce. Nevertheless, the available evidence suggests that cuticular wax traits within a species can vary substantially across different habitats. For example, Bueno et al. (2019a) found that *Quercus coccifera* trees growing in Mediterranean climate deposited 2.68‐fold more wax compared to those growing in temperate regions. Dodd et al. (1998) further reported pronounced variation in the alkane chain‐length distribution among natural populations of *Austrocedrus chilensis* across Chile and Argentina, with populations from warmer and drier Mediterranean regions tending to exhibit a greater proportion of longer‐chain hydrocarbons than populations from more mesic environments. Likewise, Li et al. (2019) observed considerable intraspecific variation in cuticular wax amount and composition among 59 natural populations of *Leymus chinensis*, but without identifying clear trends across the sampling locations. An important limitation of the currently available literature is that comparatively little is known about how different environmental drivers interact to influence wax biosynthesis. Interactions may be additive, synergistic or antagonistic. For example, light quantity consistently promotes wax accumulation across many species (Section 3.1), whereas temperature effects are comparatively weak and species‐dependent (Section 3.3). Whether changes in temperature amplify, attenuate or are completely overridden by light‐induced wax accumulation remains still largely unresolved. Similar uncertainties yet exist for interactions involving UV‐B radiation, atmospheric humidity, water scarcity or salinity.

Beyond affecting wax quantity and composition, multifactorial environments may also influence the three‐dimensional organisation of epicuticular waxes on the leaf surface. While the self‐assembly of waxes into diverse three‐dimensional structures is suggested to largely depend on the chemical wax composition (Barthlott et al. 1998; Koch and Barthlott 2006), changes in temperature and relative humidity were previously reported to modify epicuticular crystal morphology (Whitecross and Armstrong 1972; Baker 1974; Reed and Tukey 1982; Gauvrit and Gaillardon 1991). It is therefore conceivable that complex environments can contribute to establishing the final leaf surface topography.

## 4 Cuticular Wax Quantity and Its Implications for Residual (Cuticular) Transpiration

Although the amount of cutin is also known to change in response to the environment (e.g., Martin et al. 2017; He et al. 2019; Bueno et al. 2019a; Shellakkutti et al. 2022), the cuticular wax fraction establishes the pivotal barrier for water loss. Consequently, the demonstrated quantitative and qualitative changes in cuticular wax, rather than alterations in cutin amounts, were considered to be of higher biological relevance for the transpirational properties of plant cuticles. It is often intuitively assumed that increased cuticular wax loads should logically reduce residual (cuticular) transpiration by impeding water diffusion across the cuticle. Indeed, Fick's first law of diffusion postulates that the diffusion flux of a given substance (*J*) is proportional to the concentration gradient (*dC*) and the diffusion coefficient (*D*), but inversely proportional to the distance over which diffusion occurs (*dx*):

$$J=-D\frac{\mathrm{dC}}{\mathrm{dx}}.$$

When applied to the system of plant cuticles, this law suggests that a thicker (i.e., as a consequence of increased wax accumulation) cuticular wax layer (*dx*) should reduce the cuticular water permeability by increasing the distance that water molecules must traverse in order to escape the leaf interior. From this purely physicochemical perspective, a negative correlation between the cuticular wax amount and residual (cuticular) transpiration is a perfectly reasonable hypothesis. Indeed, several past studies claim that this holds true, allegedly demonstrating that increased wax amounts reduce residual (cuticular) transpiration and vice versa (e.g., reviewed by Hasanuzzaman et al. 2023; Table S1). While these observations seem to point towards a direct functional link between absolute wax amounts and the cuticular water barrier, we do hypothesise that the drawn conclusions might often be influenced by overlooked methodological biases that were discussed in Section 2 (refer also to the Supporting Information S1: Section S2). When restricting the literature to studies that meet the defined inclusion criteria, the markedly reduced dataset suggests that this relationship is not as straightforward as previously assumed (Tables 2 and 3).

### 4.1 Increased Wax Loads

In Table 2, the effects of changes in leaf surface‐area‐normalised wax amounts on the residual (cuticular) transpiration (expressed as permeance) are summarised. The compiled data clearly support the hypothesis that increased wax amounts do not necessarily translate into an enhanced water barrier function. In several cases, a pronounced increase in the cuticular wax amount had no measurable effect on the corresponding permeance. For instance, *P. × canescens* cultivated outdoors deposited 12.69‐fold more wax compared to ex vitro developed leaves of young poplars in a growth chamber (17.51 vs. 1.38 µg cm^−2^) yet exhibited nearly identical permeances (Grünhofer et al. 2022). In *Q. coccifera*, leaves from Mediterranean climates had 2.68‐fold higher wax amounts than those from temperate conditions (34.90 vs. 13.00 µg cm^−2^), but their permeance remained unchanged (Bueno et al. 2019a). Similarly, high light exposure led to a substantial increase in wax accumulation in *V. radiata* (4.64‐fold, 0.44 vs. 2.04 µg cm^−2^), *S. lycopersicum* (2.44‐fold, 1.47 vs. 3.59 µg cm^−2^), *Z. mays* (2.30‐fold, 12.07 vs. 27.80 µg cm^−2^) and *H. vulgare* (1.67‐fold, 9.72 vs. 16.23 µg cm^−2^), but these changes did not induce measurable reductions in permeance in any of the investigated species (Herzig et al. 2025). In tomato, surprisingly, even a gradual increase in residual (cuticular) transpiration with increasing wax amount was reported (up to around threefold) (Herzig et al. 2025), although a certain higher stomatal contribution as a consequence of high light exposure cannot be ruled out. In two further barley cultivars, simulating natural drought by inducing osmotic stress led to slightly increased wax loads (1.20‐ and 1.31‐fold, respectively) without affecting the respective permeances (Shellakkutti et al. 2022).

### 4.2 Decreased Wax Loads

The reverse conclusion of more wax strengthening the cuticular water barrier would be the hypothesis of less wax weakening it. However, a natural reduction in the cuticular wax amount did not uniformly increase the residual (cuticular) transpiration in the compiled data (Table 2). A seasonal comparison in different poplar species (*P. trichocarpa*, *P. euphratica* and two *P. × canescens* cultivars) revealed that cuticular wax deposits on leaves sampled in late summer were 0.75‐ to 0.41‐fold of their spring counterparts, yet instead of showing increased water loss rates, three of the four investigated genotypes even exhibited a reduced permeance to approximately 0.5‐fold of the initial value (Grünhofer et al. 2021). Further, in the *D. caryophyllus* cultivar Master, older leaves (comparing two flowering stages) showed a reduction of the cuticular wax to 0.53‐fold of their younger counterparts, which did not affect permeance (Cheng et al. 2023). Table 2 only contains one study where a decrease in wax amount coincided with an increase in permeance: a genetically engineered elongase knock‐out mutant in barley (*cer‐ye.267*, a knock‐out of *HvKCS1*; Lundqvist and Lundqvist 1988), in which the wax amounts were biotechnologically reduced to 0.30‐fold of wildtype levels (11.50 vs. 3.40 µg cm^−2^; Müller et al. 2023). However, within the same study, an additional mutant (*cer‐za.227*, a knock‐out of *HvFAR1*; Lundqvist and Lundqvist 1988) with a similar reduction in wax amount (11.50 vs. 3.20 µg cm^−2^; Müller et al. 2023) did not exhibit a significantly decreased permeance, suggesting that alterations beyond the sole wax quantity must have been responsible for the elevated permeance in *cer‐ye.267*.

### 4.3 Minimum Effective Wax Amount

One possible explanation for the weak correlation between cuticular wax amount and permeance is the postulated existence of a genetically defined ‘minimum effective amount’ of cuticular wax required to maintain an intact water barrier (Jordan et al. 1984; Grünhofer and Schreiber 2023) and that additional wax deposits exceeding this threshold would not further reduce transpiration. In fact, from a purely physical perspective, already a single continuous monolayer of very‐long‐chain (C34–C40) fatty alcohols could provide sufficient resistance to account for the low permeances observed in many cuticles (Schreiber and Schönherr 2009). However, plant cuticles are dynamic biological structures exposed to mechanical strain, growth‐related surface expansion and environmental perturbations, which could easily damage such a fragile monolayer. The actual biological minimum effective amount would therefore correspond to the wax coverage necessary to maintain a robust water barrier under varying external conditions and developmental stages. Cuticular wax amounts as little as 1 µg cm^−2^ were found to establish a fully functional water barrier in annual herbs (*A. thaliana;* Sadler et al. 2016) and in perennial trees (*P*. × *canescens*; Grünhofer et al. 2022). Considerably lower wax amounts were reported in bryophytic species, reaching values as little as 0.1 µg cm^−2^ in liverworts and even 0.02 µg cm^−2^ in mosses (Matos et al. 2021). Interestingly, these species rely largely on surface diffusion for gas exchange, as their gametophyte lacks stomata (Kong et al. 2020). Although reliable permeance‐based transpiration measurements are not available, researchers have postulated that such low amounts do not establish a fully sealed water barrier, leaving the cuticle sufficiently permeable for gas exchange (Buda et al. 2013). Instead of preventing desiccation through a highly impermeable cuticle, bryophytes have adopted a poikilohydric lifestyle and tolerate extensive dehydration (Morales‐Sánchez et al. 2022). Therefore, the deposition of a robust minimum effective wax amount might be a key adaptation associated with the evolution of homoiohydry in terrestrial plants. Considering the broad diversity of vascular land plants and the high variability in overall growth dynamics, rates of surface expansion, cuticle architecture, and the selective pressures imposed by their habitat, the required minimum wax amount likely differs between species. For example, it appears that xerophytic species growing in warm and arid climates tend to deposit more cuticular wax than temperate species (Schreiber and Riederer 1996b), but whether this also reflects higher minimum effective amounts remains speculative. Overall, the data compiled in Table 2 suggest that environmental changes do not reduce the cuticular wax loads below such an effective amount in modern angiosperms, which could readily explain why naturally reduced wax loads did not increase the residual (cuticular) transpiration in any case. Nonetheless, it is conceivable that a severe artificial reduction in cuticular wax loads through genetic engineering, contrasting natural modifications, can reduce the wax coverage below the critical minimum amount, which might indeed impair the cuticular water barrier (as, e.g., suggested in Bourdenx et al. 2011; Seo et al. 2011; Seo and Park 2011; He et al. 2022; Mao et al. 2012, although robust permeance estimations are lacking).

### 4.4 Summary

We conclude from the available data that naturally increased wax loads do not inherently decrease the residual (cuticular) transpiration (Section 4.1), while naturally decreased wax amounts do not necessarily increase it (Section 4.2). Even when disregarding the ‘comparing apples to oranges’ argument (Subsection S2.1) for a moment and thus directly comparing the compiled cuticular wax amounts and their corresponding permeance values across multiple species and environmental conditions (Figure 1), still no clear correlation can be observed: the conclusive examined data (Tables 2 and 3) span a broad range of wax quantities from approximately 1 to > 40 µg cm^−2^, but the vast majority of permeance values cluster within a relatively narrow range between 0.5 and 2.7 × 10^−9^ m s^−1^. Remarkably, when now being able to compare the entirety of permeance values across species (Figure 1) instead of just directional relations within species (Tables 2 and 3), a clear set of ‘outstanding outliers’ (data points 1–4 and 14–17 of Figure 1) emerges. On the one hand, in the case of *D. caryophyllus* (data points 1–4), differently aged leaves (older leaves are prone to stomatal malfunction) were compared with one another (Cheng et al. 2023). On the other hand, as has already been reasoned in the original publication investigating equally aged leaves grown in spring and late summer (Grünhofer et al. 2021), *P. trichocarpa* and *P. euphratica* (data points 14–17) both represent species infamous for incomplete stomatal closure. It is thus reasonable to assume that in both studies, the permeance changes might reflect altered stomatal leakiness (as a consequence of leaf age or seasonality) rather than true cuticular wax effects. Taken together, these considerations again underscore the necessity of accounting for (maximum) stomatal closure on a case‐specific basis when working with whole leaves and further emphasise the importance of contextualising obtained residual (cuticular) permeance data with true cuticular permeance values whenever possible by measuring astomatous cuticles or hypostomatous leaf discs mounted to transpiration chambers.

**Figure 1:**
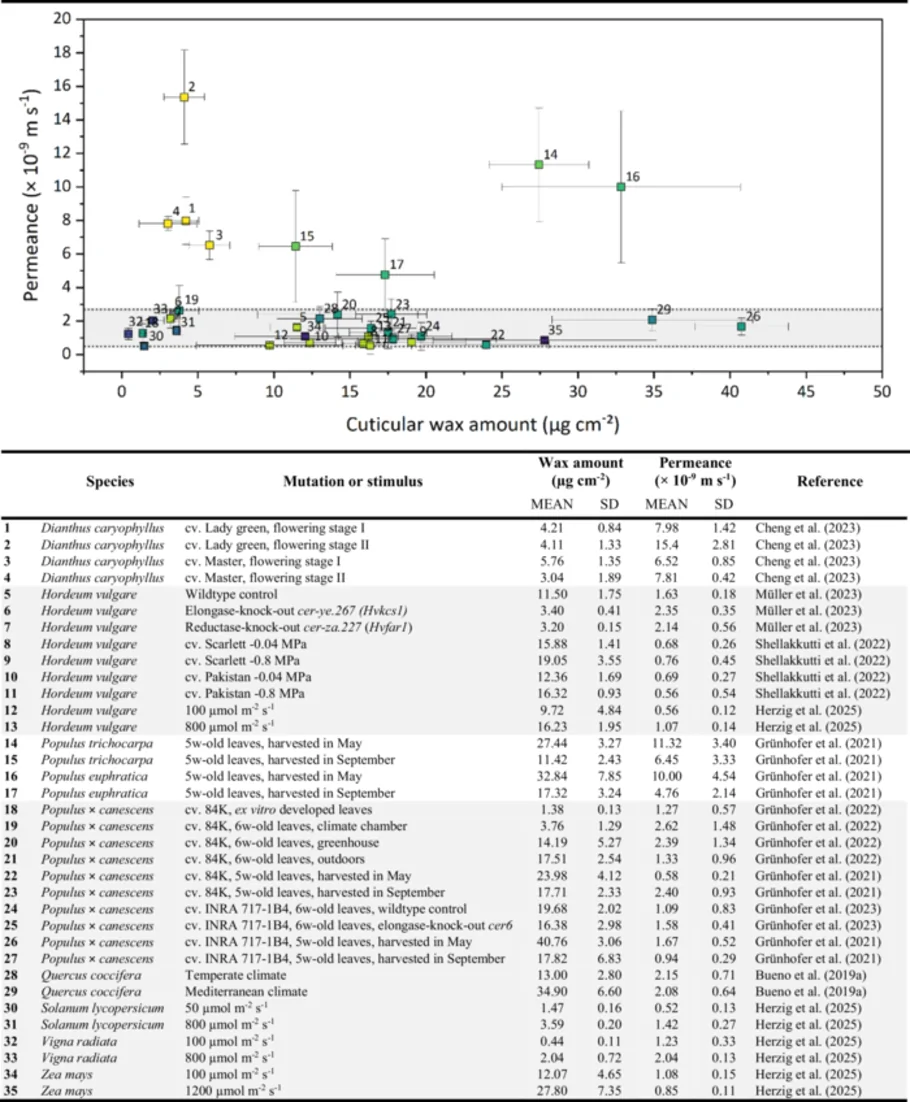
Relationship between surface‐area‐normalised absolute cuticular wax amounts and residual (cuticular) transpiration of leaves. To ensure comparability across the compiled data and provide high‐quality results, only research that employed state‐of‐the‐art analytical methods in combination with permeance measurements was included. The graph visualises the data summarised in Tables 2 and 3 and was plotted based on means and standard deviations obtained from the respective publication. Except for a few outliers (data points 1–4 and 14–17), the majority of reported permeance values cluster within 0.5–2.7 × 10^−9^ m s^−1^. Each species is shown in a different colour.

Despite the obvious lack of a correlation between cuticular wax amount and cuticular permeability within the natural physiological range, it appears possible that humanly engineered decreased cuticular wax mutants can exhibit elevated water loss, which led us to hypothesise that an artificial interference with specific steps of wax biosynthesis might compromise the cuticular water barrier in yet unrecognised ways (as will be further discussed in Section 6), whereas naturally induced reductions presumably would not.

## 5 Cuticular Wax Quality and Its Implications for Residual (Cuticular) Transpiration

Given that the cuticular water barrier efficiency apparently cannot be predicted from the sheer wax quantity alone, attention naturally turns to its composition: Could the chemical make‐up of cuticular wax represent a more determinant factor for cuticular water retention than previously assumed? Whereas the effect of cuticular wax quantity on the residual (cuticular) transpiration has been investigated in numerous studies incorporating state‐of‐the‐art methodologies (Table 2), comparatively few have yet addressed the role of cuticular wax composition in leaves (Table 3).

### 5.1 Compound Classes

Knowing that especially very‐long‐chain aliphatic components establish the cuticles' water barrier (Jetter and Riederer 2016; Seufert et al. 2021), efforts have been made to identify the contributions of individual aliphatic compound classes to transpirational properties of leaf cuticles: For example, a model designed by Bueno et al. (2019b) suggests that cuticular wax is organised in densely packed crystalline domains (formed by aliphatic carbon chains) which are essentially impermeable to water and that these domains are outlined by amorphous regions (formed by functional groups). According to their hypothesis, very‐long‐chain ester dimers could intercalate between neighbouring crystalline domains, effectively ‘bridging’ them together. This architecture was predicted to enhance cuticular stability, increase hydrophobicity and thereby reduce the cuticular water permeability based on comparisons between the water‐saver species *Phoenix dactylifera* and the water‐spender species *Citrullus colocynthis*. However, recent experimental approaches rather focusing on a single species (Table 3) also indicate that a rich ester abundance alone does not seem to predict the cuticular water barrier efficiency: The cuticular wax of a *cer6* mutant in *P*. × *canescens*, presenting wildtype‐like total wax amounts, was composed of > 90% ester dimers. However, instead of showing reduced residual (cuticular) transpiration as hypothesised according to the model of Bueno et al. (2019b), it exhibited an almost 1.5‐fold increased permeance compared to its wildtype counterpart (measured either with whole leaves or leaf discs mounted into transpiration chambers, only measuring the astomatous adaxial leaf side, thus in combination fully excluding any stomatal contribution in this case; Grünhofer et al. 2023).

While investigating the role of esters has yielded contradicting outcomes, research addressing the impact of other wax constituents on the cuticular water barrier is even more scarce. Cheng et al. (2023) demonstrated that in the *D. caryophyllus* cultivar Lady green, leaf ageing from flowering stage I to II was characterised by a + 17 pp increase in the relative portion of alcohols but unchanged total wax amounts, coinciding with an almost 2‐fold higher permeance (Table 3). This suggests that a substantial increase in the portion of alcohols might enhance the residual (cuticular) transpiration. However, considering the proposed impaired stomatal closure, especially in older leaves of this species, resulting in higher permeances as a consequence of increased stomatal contribution (Subsection 4.3, Figure 1), it remains to be verified whether a similarly pronounced increase in alcohols would have comparable effects in other species. To resolve this, future investigations should ideally focus on same‐aged leaves to minimise developmental influences on stomatal functionality.

Moreover, previous research has indicated that 5‐week‐old *P. trichocarpa* leaves sampled in spring and late summer exhibited an increase in the relative portion of alkanes by +15 pp alongside a decrease in permeance to about 0.5‐fold, whereas other poplar species analysed in the same study displayed a similar reduction in permeance without detectable shifts in wax composition (Table 2; Grünhofer et al. 2021). As suggested by Figure 1 and by the authors of the original publication, the reduced residual (cuticular) transpiration is likely influenced by seasonal changes in stomatal behaviour (e.g., more efficient stomatal closure in late summer would reduce stomatal contribution and ultimately result in lower permeances) rather than being a direct consequence of the observed shifts in wax composition.

### 5.2 Weighted Mean Chain Length

Already over 30 years ago, Riederer and Schneider (1990) suggested that the length of aliphatic hydrocarbon chains could be even more decisive for building a transport barrier than their potential polar functional groups. They proposed that an increase in the weighted mean carbon chain length could increase the volume of the impermeable crystalline sites, which would in turn reduce the abundance of amorphous pathways and thus decrease the permeability towards water. Experimental evidence from non‐foliar organs supports this model, for instance, in mutated *S. lycopersicum* fruit cuticles (Vogg et al. 2004) or comparing vegetative to reproductive plant organs in *Rosa chinensis* (Cheng et al. 2019) or *D. caryophyllus* (Cheng et al. 2023). In all cases, elevated permeances were attributed to a decrease in the weighted mean carbon chain length. When the focus is restricted to leaves, the *P*. × *canescens cer6* mutant (Table 3), characterised by a substantial shift in weighted mean chain length from 34.1 to 40.7, surprisingly showed an increased permeance (while any residual stomatal contribution was methodologically excluded), thus opposing the initial literature‐based hypothesis (Grünhofer et al. 2023). As of now, it appears that there is no straightforward and universally applicable relationship between the weighted mean chain length and the cuticular water barrier function. Nonetheless, it should be considered that, especially in the case of the mutation‐induced shifts in chain length distribution, disturbance of the cuticular ultrastructure (see Section 6) as an unintended side effect could be responsible for the increased cuticular permeability.

### 5.3 Summary

The compiled (but still very limited) data did not support the literature‐based models on the role of either specific compound classes (Section 5.1) or the weighted mean carbon chain length (Section 5.2) in determining residual (cuticular) transpiration in leaves. However, because we assume that the permeances in *D. caryophyllus* and *P. trichocarpa* could be confounded by differentially acting stomata (a leaf age‐ or seasonality‐impacted stomatal contribution might solitarily lead to altered residual (cuticular) transpiration, as discussed in Subsection 4.3), it remains elusive to what degree these findings represent reproducible facts. Consequently, after applying our strict inclusion criteria and further treating these two possibly confounded cases with great caution, only a single dataset remains for now that can be interpreted with sufficient confidence (since stomatal contribution was accounted for as carefully as possible): a wildtype and a *cer6* mutant of *P*. × *canescens* that were part of a comprehensive experimental series aiming to clearly distinguish ‘amount effects’ from ‘composition effects’ on the residual (cuticular) transpiration (Grünhofer et al. 2022, 2023; Figure 2): The quantity‐based approach focusing on the wildtype in different environmental conditions confirmed that naturally increased cuticular wax amounts (Figure 2a) with unaltered wax composition (Figure 2b) did not affect the residual (cuticular) transpiration when considering previously published upper and lower thresholds (Figure 2c), which upholds the pattern observed across different species (Figure 1) on a robust within‐species foundation. In contrast, the quality‐based approach revealed that the transgenic *cer6* mutant with near‐identical wildtype‐like wax amounts (Figure 2d) but severely altered relative wax composition (> 90% very‐long‐chained wax esters and a consequently increased weighted mean carbon chain length, Figure 2e) exhibited a counterhypothetical 1.5‐fold increase in the residual (cuticular) transpiration, which was nonetheless still very well in the previously reported ‘natural’ range (Figure 2f).

**Figure 2:**
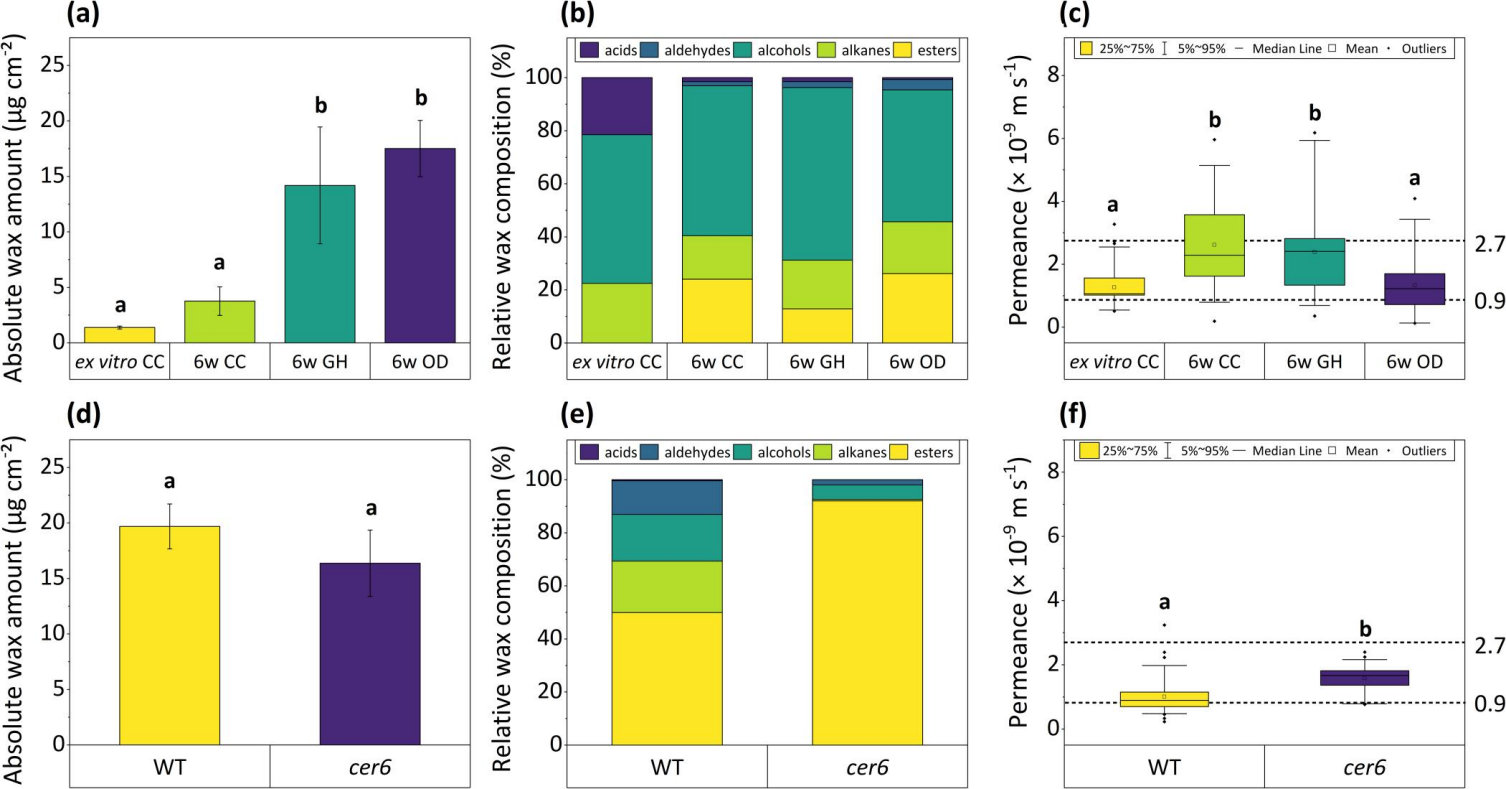
Effect of cultivation conditions (a–c) or knock‐out of a cuticular wax elongase (d–f) on the surface‐area‐normalised absolute cuticular wax amount (a, d), the relative cuticular wax composition (b, e), and the residual (cuticular) transpiration expressed as permeance (c, f) of *Populus* × *canescens* leaves. (a–c) Ex vitro climate chamber‐developed leaves compared to 6‐week‐old leaves developed in a climate chamber, a greenhouse or outdoors (Grünhofer et al. 2022). (d–f) 6‐Week‐old leaves of wildtype poplar and a *cer6* mutant cultivated in a greenhouse (Grünhofer et al. 2023). As a reference, the lowest (residual; 0.9 × 10^−9^ m s^−1^) and highest (cuticular; 2.7 × 10^−9^ m s^−1^) previously published mean residual (cuticular) transpiration rates for *P*. × *canescens* leaves are marked by dotted lines (Schreiber et al. 2006; Grünhofer et al. 2021). Different letters indicate significant differences at *p* < 0.05. CC, climate chamber; GH, greenhouse; OD, outdoors; w, weeks; WT, wildtype. [Color figure can be viewed at wileyonlinelibrary.com]

Even though these findings contradict the models proposed by Bueno et al. (2019b) and Riederer and Schneider (1990), we do agree that it might be a valuable approach to put more emphasis on how the chemical composition can shape the structural organisation of the cuticle (as also recently considered in Tiloca et al. 2026). We further hypothesise that especially biotechnological manipulation of cuticular wax biosynthesis (in contrast to ‘naturally’ resulting qualitative adaptations) might alter the structural arrangement within the cuticle, thus substantially affecting its water permeability in any yet unforeseeable direction.

## 6 Structural Heterogeneity of Leaf Cuticles

It is already well‐established that plant cuticles are not homogeneous layers, but that they exhibit a considerable heterogeneity in both structure and function (Riederer and Müller 2006; Jetter and Riederer 2016; Zeisler‐Diehl et al. 2018). In the following subsections, we will discuss two aspects of this organisational complexity that might constitute yet under‐represented factors in shaping residual (cuticular) water loss alongside cuticular wax quantity and quality.

### 6.1 Intra‐ and Epicuticular Wax

A key aspect of the cuticle's structural heterogeneity is its vertical organisation. Cuticular waxes can be subdivided into intracuticular waxes embedded within the cutin matrix and an epicuticular fraction deposited on top of the cuticle. Previous research has shown that this subdivision is not merely spatial but also functional: epicuticular waxes primarily reduce the retention of water on the leaf surface, scatter harmful radiation, and form the first line of defence against pathogens and insects (Koch and Barthlott 2006; Chiu et al. 2020). In contrast, it has been suggested that the water barrier essentially resides within a thin layer of intracuticular wax (Zeisler and Schreiber 2016; Zeisler‐Diehl et al. 2018; Müller et al. 2023). Assuming that this functional separation is broadly conserved across most species would call into question whether it is appropriate at all to treat the total cuticular wax (comprising both the intra‐ and epicuticular fraction at the same time) as a single, unified parameter when evaluating residual (cuticular) water barrier properties. For instance, it is possible that the changes in total wax amount reported in Table 2 largely reflect a response in the epicuticular fraction, but that a minimum effective amount of intracuticular wax remains unaltered. In this scenario, it would not be surprising that no reliable correlation between the total cuticular wax amount and residual (cuticular) transpiration has yet been discovered. To explore this hypothesis, more research investigating the plasticity of intra‐ versus epicuticular wax in separation would be of high interest in the future.

### 6.2 Pathways of Cuticular Water Diffusion

In addition to vertical inconsistency, the cuticle also exhibits a pronounced lateral heterogeneity. In principle, the cuticle acts as a solution‐diffusion membrane, which means that individual water molecules follow a random path across the lipophilic cutin and wax matrix (Kerstiens 1996; Schreiber and Riederer 1996a; Riederer and Schreiber 2001). Accumulating evidence has confirmed that, in addition to a lipophilic route, a secondary, more hydrophilic pathway across the cuticle must exist that promotes the movement of water and larger ionic molecules (Schreiber 2006; Remus‐Emsermann et al. 2011). This hydrophilic pathway is presumably established by ‘weak spots’ in the membrane that arise from the hydration of polar functional groups within the (intracuticular) wax matrix (Chamel et al. 1991). These spots have been previously described as ‘aqueous pores’ (Schönherr 2000, 2006; Schönherr and Schreiber 2004; Schlegel et al. 2005) or recently also as a ‘dynamic aqueous continuum’ (Fernandez et al. 2017), and their potential role in cuticular water resistance has gained increasing attention: Because water molecules are small and uncharged, they can traverse the cuticle over the lipophilic (cutin and wax matrix) and the hydrophilic (aqueous domains) pathway in parallel, but their relative contribution to total permeance remains uncertain. Selective blockage of the hydrophilic pathway through the formation of insoluble AgCl precipitates was shown to reduce the cuticular permeance between 0.85‐ and 0.36‐fold (comparing 13 different species; Schreiber et al. 2006). Conversely, increasing the abundance of aqueous domains might be able to facilitate water permeance by a similar factor. The elevated permeances observed in independent cuticular wax mutants (Tables 2 and 3) might be related to an unintentionally promoted formation of such domains as a side effect of genetic manipulation, ultimately resulting in elevated residual (cuticular) water permeabilities.

## 7 Concluding Remarks and Perspectives

The previous discussions based on a thoroughly selected dataset have provided valuable insight into the environmental regulation of cuticular wax in modern angiosperms (Section 3), how either quantitative (Section 4) or qualitative (Section 5) alterations relate to residual (cuticular) transpiration, and offered hypothetical explanations for the observed (or even non‐existent) patterns (Section 6). The presented reassessment revealed that some misconceptions still prevail, and intending to move beyond comprehensible yet certainly oversimplified interpretations led us to draw the following conclusions and perspectives about the dynamics of cuticular wax and its relation to residual (cuticular) transpiration:

i. Environmental stimuli can strongly influence the surface‐area‐normalised cuticular wax amount, while their effect on the relative composition appears to be more case‐specific (Section 3 and Table 1). Light quantity and drought were shown to be strong and uniform drivers of cuticular wax deposition across independent species, while the effects of temperature, relative humidity and NaCl exposure were less pronounced. However, the degree of plasticity in cuticular wax amount, especially in cuticular wax composition, differed between species and appeared to depend on the precise experimental setup, including cultivation conditions, treatment duration and intensity, and leaf developmental state. These findings suggest that environmental responses are tightly controlled on a genetic level, but the precise underlying molecular pathways, and how their regulation appears to vary between species and contexts, remain to be further unravelled in the future. In addition, the limited availability of multifactorial studies currently restricts our ability to extrapolate findings from controlled single‐factor experiments to natural ecosystems. Future research should therefore aim to combine environmental variables in experimental designs and integrate chemical and structural analyses in order to better understand how cuticular waxes respond to realistic environmental scenarios.
ii. The compiled results demonstrated that within the natural physiological range of variation in wax quantity, the cuticular wax amount does not seem to predict residual (cuticular) transpiration of leaves (Section 4, Table 2 and Figures 1 and 2), which reinforces the assumption that the intuitive ‘more cuticular wax equals lower rates of transpiration’ hypothesis is not supported once methodological pitfalls are accounted for (Section 2). We hypothesise that the lack of a correlation between total cuticular wax amounts and residual (cuticular) transpiration could either be due to a stable effective minimum amount of intracuticular wax that remains unaltered even when epicuticular wax loads change, thus providing a steady water barrier, that hydrophilic domains within the lipophilic cuticle allow for a constant water movement even when wax deposits accumulate in response to external cues (Section 6), or a combination of both. While these are the first evidence‐based assumptions to explain the compiled findings, they might act together with further and potentially yet unidentified ultrastructural reorganisations. To clarify the underlying mechanisms, it would be valuable to separately examine intra‐ and epicuticular wax fractions, as well as further investigate the contribution of hydrophilic pathways of diffusion (for instance, through selective blockage using AgCl precipitates) in future studies.
iii. While the effect of environmentally driven ‘natural’ changes appears to be limited, ‘artificial’ reduction of cuticular wax loads through genetic manipulation might affect the cuticular water permeability (Section 4). However, since studies investigating biotechnologically engineered mutants with reduced cuticular wax loads also scarcely incorporate permeance estimations, we can currently only speculate that an artificial reduction of cuticular wax below a critical minimum effective amount likely impairs the cuticular water barrier. Besides potentially disrupting an effective minimum cuticular wax amount, it was proposed that genetic engineering itself might unintentionally confound the cuticular ultrastructure (e.g., by disorganising the structure of an effective intracuticular wax layer or by increasing the abundance of aqueous pores, Section 6). Thus, it could become relevant for future research to interpret the permeability effect of environmentally versus biotechnologically induced modifications of the cuticular wax amount in strict separation.
iv. The relative wax composition might play a more pivotal role in mediating cuticular water barrier properties than the sheer wax amount (Section 5, Table 3 and Figure 2). While the current research generally supports the hypotheses of shifts in compound class ratios or chain length distribution affecting residual (cuticular) transpiration, it was not possible to confirm any consistent relationships based on our very limited dataset. As of now, the first genetic engineering approaches have even yielded surprising results, contrary to literature‐based expectations. This further supported the assumption that humanly introduced manipulation of the cuticular wax biosynthesis could induce unintended side effects compromising cuticular integrity, thereby altering water loss properties (Section 6). The precise underlying effects, however, remain to be elucidated in future studies.

## Conflicts of Interest

The authors declare no conflicts of interest.

## Supporting information


Supporting File

## Data Availability

Data sharing not applicable to this article as no datasets were generated or analysed during the current study.

## References

[pce70714-bib-0001] Baker, E. A. 1974. “The Influence of Environment on Leaf Wax Development in *Brassica oleracea* var. *gemmifera* .” New Phytologist 73, no. 5: 955–966. 10.1111/j.1469-8137.1974.tb01324.x.

[pce70714-bib-0002] Barthlott, W. , C. Neinhuis , D. Cutler , et al. 1998. “Classification and Terminology of Plant Epicuticular Waxes.” Botanical Journal of the Linnean Society 126, no. 3: 237–260. 10.1111/j.1095-8339.1998.tb02529.x.

[pce70714-bib-0003] Bourdenx, B. , A. Bernard , F. Domergue , et al. 2011. “Overexpression of Arabidopsis ECERIFERUM1 Promotes Wax Very‐Long‐Chain Alkane Biosynthesis and Influences Plant Response to Biotic and Abiotic Stresses.” Plant Physiology 156, no. 1: 29–45. 10.1104/pp.111.172320. PMC3091054 21386033

[pce70714-bib-0004] Buda, G. J. , W. J. Barnes , E. A. Fich , et al. 2013. “An ATP Binding Cassette Transporter Is Required for Cuticular Wax Deposition and Desiccation Tolerance in the Moss *Physcomitrella patens* .” Plant Cell 25, no. 10: 4000–4013. 10.1105/tpc.113.117648. PMC3877811 24163310

[pce70714-bib-0005] Budke, J. M. , B. Goffinet , and C. S. Jones . 2011. “A Hundred‐Year‐Old Question: Is the Moss Calyptra Covered by a Cuticle? A Case Study of *Funaria hygrometrica* .” Annals of Botany 107, no. 8: 1279–1286. 10.1093/aob/mcr079. PMC3101145 21486928

[pce70714-bib-0006] Bueno, A. , A. Alfarhan , K. Arand , et al. 2019b. “Effects of Temperature on the Cuticular Transpiration Barrier of Two Desert Plants With Water‐Spender and Water‐Saver Strategies.” Journal of Experimental Botany 70, no. 5: 1613–1625. 10.1093/jxb/erz018. PMC6416792 30715440

[pce70714-bib-0007] Bueno, A. , D. Sancho‐Knapik , E. Gil‐Pelegrín , et al. 2019a. “Cuticular Wax Coverage and Its Transpiration Barrier Properties in *Quercus coccifera* L. Leaves: Does the Environment Matter?.” Tree Physiology 40, no. 7: 827–840. 10.1093/treephys/tpz0110. 31728539

[pce70714-bib-0008] Burghardt, M. 2003. “Ecophysiological Relevance of Cuticular Transpiration of Deciduous and Evergreen Plants in Relation to Stomatal Closure and Leaf Water Potential.” Journal of Experimental Botany 54, no. 389: 1941–1949. 10.1093/jxb/erg195. 12815029

[pce70714-bib-0009] Burghardt, M. , A. Burghardt , J. Gall , C. Rosenberger , and M. Riederer . 2008. “Ecophysiological Adaptations of Water Relations of *Teucrium chamaedrys* L. to the Hot and Dry Climate of Xeric Limestone Sites in Franconia (Southern Germany).” Flora—Morphology, Distribution, Functional Ecology of Plants 203, no. 1: 3–13. 10.1016/j.flora.2007.11.003.

[pce70714-bib-0010] Cameron, K. D. , M. A. Teece , and L. B. Smart . 2006. “Increased Accumulation of Cuticular Wax and Expression of Lipid Transfer Protein in Response to Periodic Drying Events in Leaves of Tree Tobacco.” Plant Physiology 140, no. 1: 176–183. 10.1104/pp.105.069724. PMC1326042 16361524

[pce70714-bib-0011] Chamel, A. , M. Pineri , and M. Escoubes . 1991. “Quantitative Determination of Water Sorption by Plant Cuticles.” Plant Cell & Environment 14, no. 1: 87–95. 10.1111/j.1365-3040.1991.tb01374.x.

[pce70714-bib-0012] Chen, M. , X. Zhu , Y. Zhang , et al. 2020. “Drought Stress Modify Cuticle of Tender Tea Leaf and Mature Leaf for Transpiration Barrier Enhancement Through Common and Distinct Modes.” Scientific Reports 10, no. 1: 6696. 10.1038/s41598-020-63683-4. PMC7174317 32317754

[pce70714-bib-0013] Cheng, G. , C. Ye , J. Zhang , H. Li , Y. Jiang , and X. Duan . 2023. “Water Permeability of Different Aerial Tissues of Carnations Is Related to Cuticular Wax Composition.” Physiologia Plantarum 175, no. 2: e13883. 10.1111/ppl.13883. 36840510

[pce70714-bib-0108] Cheng, G. , H. Huang , L. Zhou , S. He , Y. Zhang , and X. Cheng . 2019. “Chemical Composition and Water Permeability of the Cuticular Wax Barrier in Rose Leaf and Petal: A Comparative Investigation.” Plant Physiology and Biochemistry 135: 404–410. 10.1016/j.plaphy.2019.01.006. 30635221

[pce70714-bib-0014] Chiu, Y.‐C. , C. Shen , M. W. Farnham , and K.‐M. Ku . 2020. “Three‐Dimensional Epicuticular Wax on Plant Surface Reduces Attachment and Survival Rate of Salmonella During Storage.” Postharvest Biology and Technology 166: 111197. 10.1016/j.postharvbio.2020.111197.

[pce70714-bib-0015] Conner, L. N. , and A. J. Conner . 1984. “Comparative Water Loss From Leaves of *Solanum laciniatum* Plants Cultured In Vitro and In Vivo.” Plant Science Letters 36, no. 3: 241–246. 10.1016/0304-4211(84)90176-7.

[pce70714-bib-0016] de Araújo Silva, M. M. , L. T. Ferreira , F. M. T. de Vasconcelos , et al. 2021. “Water Stress‐Induced Responses in the Growth, Cuticular Wax Composition, Chloroplast Pigments and Soluble Protein Content, and Redox Metabolism of Two Genotypes of *Ricinus communis* L.” Journal of Plant Growth Regulation 40, no. 1: 342–352. 10.1007/s00344-020-10103-6.

[pce70714-bib-0017] Dodd, R. S. , Z. A. Rafii , and A. B. Power . 1998. “Ecotypic Adaptation in *Austrocedrus chilensis* in Cuticular Hydrocarbon Composition.” New Phytologist 138, no. 4: 699–708. 10.1046/j.1469-8137.1998.00142.x.

[pce70714-bib-0018] Even, M. , M. Sabo , D. Meng , T. Kreszies , L. Schreiber , and W. Fricke . 2018. “Night‐Time Transpiration in Barley (*Hordeum vulgare*) Facilitates Respiratory Carbon Dioxide Release and Is Regulated During Salt Stress.” Annals of Botany 122, no. 4: 569–582. 10.1093/aob/mcy084. PMC6153476 29850772

[pce70714-bib-0109] Fernández, V. , H. A. Bahamonde , J. Javier Peguero‐Pina , et al. 2017. “Physico‐Chemical Properties of Plant Cuticles and Their Functional and Ecological Significance.” Journal of Experimental Botany 68, no. 19: 5293–5306. 10.1093/jxb/erx302. 28992247

[pce70714-bib-0019] Fricke, W. , G. Akhiyarova , W. Wei , et al. 2006. “The Short‐Term Growth Response to Salt of the Developing Barley Leaf.” Journal of Experimental Botany 57, no. 5: 1079–1095. 10.1093/jxb/erj095. 16513814

[pce70714-bib-0020] Gauvrit, C. , and P. Gaillardon . 1991. “Effect of Low Temperatures on 2,4‐D Behaviour in Maize Plants.” Weed Research 31, no. 3: 135–142. 10.1111/j.1365-3180.1991.tb01752.x.

[pce70714-bib-0021] Go, Y. S. , H. Kim , H. J. Kim , and M. C. Suh . 2014. “Arabidopsis Cuticular Wax Biosynthesis Is Negatively Regulated by the DEWAX Gene Encoding an AP2/ERF‐Type Transcription Factor.” Plant Cell 26, no. 4: 1666–1680. 10.1105/tpc.114.123307. PMC4036578 24692420

[pce70714-bib-0025] Grünhofer, P. , and L. Schreiber . 2023. “Humboldt Review: Cutinized and Suberized Barriers in Leaves and Roots: Similarities and Differences.” Journal of Plant Physiology 282: 153921. 10.1016/j.jplph.2023.153921. 36780757

[pce70714-bib-0022] Grünhofer, P. , L. Herzig , and L. Schreiber . 2021. “Leaf Morphology, Wax Composition, and Residual (Cuticular) Transpiration of Four Poplar Clones.” Trees 36, no. 2: 645–658. 10.1007/s00468-021-02236-2.

[pce70714-bib-0024] Grünhofer, P. , L. Herzig , Q. Zhang , et al. 2024. “Changes in Wax Composition but Not Amount Enhance Cuticular Transpiration.” Plant, Cell & Environment 47, no. 1: 91–105. 10.1111/pce.14719. 37718770

[pce70714-bib-0023] Grünhofer, P. , L. Herzig , S. Sent , V. V. Zeisler‐Diehl , and L. Schreiber . 2022. “Increased Cuticular Wax Deposition Does Not Change Residual Foliar Transpiration.” Plant, Cell & Environment 45, no. 4: 1157–1171. 10.1111/pce.14274. 35102563

[pce70714-bib-0026] Grünhofer, P. , P. D. Thangamani , L. Schreiber , and T. Kreszies . 2025. “Sodium Chloride Enhances Suberization in Seminal Roots but Does Not Affect Cutinized Leaf Barriers in Cultivated and Wild Barley.” Planta 262, no. 2: 28. 10.1007/s00425-025-04743-9. PMC12167721 40518454

[pce70714-bib-0027] Guo, Y. , J. J. Li , L. Busta , and R. Jetter . 2018. “Coverage and Composition of Cuticular Waxes on the Fronds of the Temperate Ferns *Pteridium aquilinum*, *Cryptogramma crispa*, *Polypodium glycyrrhiza*, *Polystichum munitum* and *Gymnocarpium dryopteris* .” Annals of Botany 122, no. 4: 555–568. 10.1093/aob/mcy078. PMC6153475 30252045

[pce70714-bib-0028] Hasanuzzaman, M. , M. Zhou , and S. Shabala . 2023. “How Does Stomatal Density and Residual Transpiration Contribute to Osmotic Stress Tolerance?.” Plants 12, no. 3: 494. 10.3390/plants12030494. PMC9919688 36771579

[pce70714-bib-0029] He, J. , C. Li , N. Hu , et al. 2022. “ECERIFERUM1‐6A Is Required for the Synthesis of Cuticular Wax Alkanes and Promotes Drought Tolerance in Wheat.” Plant Physiology 190, no. 3: 1640–1657. 10.1093/plphys/kiac394. PMC9614490 36000923

[pce70714-bib-0030] He, J. , S. Tang , D. Yang , et al. 2019. “Chemical and Transcriptomic Analysis of Cuticle Lipids Under Cold Stress in *Thellungiella salsuginea* .” International Journal of Molecular Sciences 20, no. 18: 4519. 10.3390/ijms20184519. PMC6770325 31547275

[pce70714-bib-0031] Herzig, L. , K. Uellendahl , Y. Malkowsky , L. Schreiber , and P. Grünhofer . 2025. “In a Different Light: Irradiation‐Induced Cuticular Wax Accumulation Fails to Reduce Cuticular Transpiration.” Plant, Cell & Environment 48, no. 5: 3632–3646. 10.1111/pce.15376. PMC11963476 39806923

[pce70714-bib-0032] Huang, L. , Q. Xiao , X. Zhao , et al. 2020. “Responses of Cuticular Waxes of Faba Bean to Light Wavelengths and Selection of Candidate Genes for Cuticular Wax Biosynthesis.” Plant Genome 13, no. 3: e20058. 10.1002/tpg2.20058. PMC12807296 33124766

[pce70714-bib-0033] IPCC . 2023. Climate Change 2023: Synthesis Report. Contribution of Working Groups I, II and III to the Sixth. Geneva, Switzerland.

[pce70714-bib-0034] Jenks, M. A. , L. Andersen , R. S. Teusink , and M. H. Williams . 2001. “Leaf Cuticular Waxes of Potted Rose Cultivars as Affected by Plant Development, Drought and Paclobutrazol Treatments.” Physiologia Plantarum 112, no. 1: 62–70. 10.1034/j.1399-3054.2001.1120109.x. 11319016

[pce70714-bib-0035] Jetter, R. , and M. Riederer . 2016. “Localization of the Transpiration Barrier in the Epi‐ and Intracuticular Waxes of Eight Plant Species: Water Transport Resistances Are Associated With Fatty Acyl Rather Than Alicyclic Components.” Plant Physiology 170, no. 2: 921–934. 10.1104/pp.15.01699. PMC4734581 26644508

[pce70714-bib-0036] Jordan, W. R. , P. J. Shouse , A. Blum , F. R. Miller , and R. L. Monk . 1984. “Environmental Physiology of Sorghum. II. Epicuticular Wax Load and Cuticular Transpiration.” Crop Science 24, no. 6: 1168–1173. 10.2135/cropsci1984.0011183X002400060038x.

[pce70714-bib-0038] Kerstiens, G. 1994. “Effects of Low Light Intensity and High Air Humidity on Morphology and Permeability of Plant Cuticles, With Special Respect to Plants Cultured In Vitro.” In Physiology, Growth and Development of Plants in Culture, edited by P. J. Lumsden , J. R. Nicholas , and W. J. Davies , 132–142. Springer.

[pce70714-bib-0037] Kerstiens, G. 1996. “Cuticular Water Permeability and Its Physiological Significance.” Journal of Experimental Botany 47, no. 12: 1813–1832. 10.1093/jxb/47.12.1813.

[pce70714-bib-0039] Kim, K. S. , S. H. Park , and M. A. Jenks . 2007b. “Changes in Leaf Cuticular Waxes of Sesame (*Sesamum indicum* L.) Plants Exposed to Water Deficit.” Journal of Plant Physiology 164, no. 9: 1134–1143. 10.1016/j.jplph.2006.07.004. 16904233

[pce70714-bib-0040] Kim, K. S. , S. H. Park , D. K. Kim , and M. A. Jenks . 2007a. “Influence of Water Deficit on Leaf Cuticular Waxes of Soybean (*Glycine max* [L.] Merr.).” International Journal of Plant Sciences 168, no. 3: 307–316. 10.1086/510496.

[pce70714-bib-0041] Koch, K. , and W. Barthlott . 2006. “Plant Epicuticular Waxes: Chemistry, Form, Self‐Assembly and Function.” Natural Product Communications 1, no. 11: 1067–1072. 10.1177/1934578X0600101123.

[pce70714-bib-0042] Koch, K. , K. D. Hartmann , L. Schreiber , W. Barthlott , and C. Neinhuis . 2006. “Influences of Air Humidity During the Cultivation of Plants on Wax Chemical Composition, Morphology and Leaf Surface Wettability.” Environmental and Experimental Botany 56, no. 1: 1–9. 10.1016/j.envexpbot.2004.09.013.

[pce70714-bib-0043] Kong, L. , Y. Liu , P. Zhi , et al. 2020. “Origins and Evolution of Cuticle Biosynthetic Machinery in Land Plants.” Plant Physiology 184, no. 4: 1998–2010. 10.1104/pp.20.00913. PMC7723097 32934149

[pce70714-bib-0044] Kosma, D. K. , B. Bourdenx , A. Bernard , et al. 2009. “The Impact of Water Deficiency on Leaf Cuticle Lipids of Arabidopsis.” Plant Physiology 151, no. 4: 1918–1929. 10.1104/pp.109.141911. PMC2785987 19819982

[pce70714-bib-0046] Kunst, L. 2003. “Biosynthesis and Secretion of Plant Cuticular Wax.” Progress in Lipid Research 42, no. 1: 51–80. 10.1016/S0163-7827(02)00045-0. 12467640

[pce70714-bib-0047] Kunst, L. , and L. Samuels . 2009. “Plant Cuticles Shine: Advances in Wax Biosynthesis and Export.” Current Opinion in Plant Biology 12, no. 6: 721–727. 10.1016/j.pbi.2009.09.009. 19864175

[pce70714-bib-0048] Lewandowska, M. , A. Keyl , and I. Feussner . 2020. “Wax Biosynthesis in Response to Danger: Its Regulation Upon Abiotic and Biotic Stress.” New Phytologist 227, no. 3: 698–713. 10.1111/nph.16571. 32242934

[pce70714-bib-0049] Li, Y. , X. Hou , X. Li , et al. 2019. “Will the Climate of Plant Origins Influence the Chemical Profiles of Cuticular Waxes on Leaves of *Leymus chinensis* in a Common Garden Experiment?.” Ecology and Evolution 10, no. 1: 543–556. 10.1002/ece3.5930. PMC6972809 31988740

[pce70714-bib-0050] Lundqvist, U. , and A. Lundqvist . 1988. “Mutagen Specificity in Barley for 1580 Eceriferum Mutants Localized to 79 Loci.” Hereditas 108, no. 1: 1–12. 10.1111/j.1601-5223.1988.tb00676.x.

[pce70714-bib-0051] Lykholat, Y. V. , N. A. Khromykh , O. O. Didur , A. A. Alexeyeva , and T. Y. Lykholat . 2020. “Modification of the Epicuticular Waxes of Plant Leaves Due to Increased Sunlight Intensity.” Biosystems Diversity 28, no. 1: 29–33. 10.15421/012005.

[pce70714-bib-0052] Mao, B. , Z. Cheng , C. Lei , et al. 2012. “Wax Crystal‐Sparse leaf2, a Rice Homologue of WAX2/GL1, Is Involved in Synthesis of Leaf Cuticular Wax.” Planta 235, no. 1: 39–52. 10.1007/s00425-011-1481-1. 21809091

[pce70714-bib-0053] Martin, L. B. B. , P. Romero , E. A. Fich , D. S. Domozych , and J. K. C. Rose . 2017. “Cuticle Biosynthesis in Tomato Leaves Is Developmentally Regulated by Abscisic Acid.” Plant Physiology 174, no. 3: 1384–1398. 10.1104/pp.17.00387. PMC5490907 28483881

[pce70714-bib-0054] Matos, T. M. , D. F. Peralta , L. P. Roma , and D. Y. A. C. Santos . 2021. “The Morphology and Chemical Composition of Cuticular Waxes in Some Brazilian Liverworts and Mosses.” Journal of Bryology 43, no. 2: 129–137. 10.1080/03736687.2020.1859872.

[pce70714-bib-0055] Merced, A. , and K. S. Renzaglia . 2017. “Structure, function and Evolution of Stomata From a Bryological Perspective.” Bryophyte Diversity and Evolution 39, no. 1: 7–20. 10.11646/bde.39.1.4.

[pce70714-bib-0056] Mittler, R. 2006. “Abiotic Stress, the Field Environment and Stress Combination.” Trends in Plant Science 11, no. 1: 15–19. 10.1016/j.tplants.2005.11.002. 16359910

[pce70714-bib-0057] Morales‐Sánchez, J. Á. , K. Mark , J. P. S. Souza , and Ü. Niinemets . 2022. “Desiccation‐Rehydration Measurements in Bryophytes: Current Status and Future Insights.” Journal of Experimental Botany 73, no. 13: 4338–4361. 10.1093/jxb/erac172. 35536655

[pce70714-bib-0058] Müller, Y. , P. Patwari , T. Stöcker , et al. 2023. “Isolation and Characterization of the Gene HvFAR1 Encoding Acyl‐CoA Reductase From the cer‐za.227 Mutant of Barley (*Hordeum vulgare*) and Analysis of the Cuticular Barrier Functions.” New Phytologist 239, no. 5: 1903–1918. 10.1111/nph.19063. 37349864

[pce70714-bib-0059] Mulroy, T. W. 1979. “Spectral Properties of Heavily Glaucous and Non‐Glaucous Leaves of a Succulent Rosette‐Plant.” Oecologia 38: 349–357. 10.1007/BF00345193. 28309493

[pce70714-bib-0060] Ni, Y. , Y. J. Guo , Y. J. Guo , L. Han , H. Tang , and M. Conyers . 2012. “Leaf Cuticular Waxes and Physiological Parameters in Alfalfa Leaves as Influenced by Drought.” Photosynthetica 50, no. 3: 458–466. 10.1007/s11099-012-0055-1.

[pce70714-bib-0061] Nobel, Park S. , ed. 2020. Physicochemical and Environmental Plant Physiology. 5th ed. Academic Press.

[pce70714-bib-0062] Patwari, P. , V. Salewski , K. Gutbrod , et al. 2019. “Surface Wax Esters Contribute to Drought Tolerance in Arabidopsis.” Plant Journal 98, no. 4: 727–744. 10.1111/tpj.14269. 30729606

[pce70714-bib-0063] Plessis, A. 2023. “Abiotic Stress Experiments Need a Reality Check to Improve Translation to the Field.” Journal of Experimental Botany 74, no. 6: 1741–1744. 10.1093/jxb/erac509. PMC10049913 36626547

[pce70714-bib-0064] Poorter, H. , F. Fiorani , R. Pieruschka , et al. 2016. “Pampered Inside, Pestered Outside? Differences and Similarities Between Plants Growing in Controlled Conditions and in the Field.” New Phytologist 212, no. 4: 838–855. 10.1111/nph.14243. 27783423

[pce70714-bib-0065] Rahman, T. , M. Shao , S. Pahari , et al. 2021. “Dissecting the Roles of Cuticular Wax in Plant Resistance to Shoot Dehydration and Low‐Temperature Stress in Arabidopsis.” International Journal of Molecular Sciences 22, no. 4: 1554. 10.3390/ijms22041554. PMC7913816 33557073

[pce70714-bib-0066] Reed, D. W. , and H. B. Tukey . 1982. “Light Intensity and Temperature Effects on Epicuticular Wax Morphology and Internal Cuticle Ultrastructure of Carnation and Brussels Sprouts Leaf Cuticles.” Journal of the American Society for Horticultural Science 107, no. 3: 417–420.

[pce70714-bib-0067] Remus‐Emsermann, M. N. , S. de Oliveira , L. Schreiber , and J. H. Leveau . 2011. “Quantification of Lateral Heterogeneity in Carbohydrate Permeability of Isolated Plant Leaf Cuticles.” Frontiers in Microbiology 2: 197. 10.3389/fmicb.2011.00197. PMC3202220 22046169

[pce70714-bib-0070] Riederer, M. , and C. Müller , eds. 2006. Annual Plant Reviews Vol. 23: Biology of the Plant Cuticle. Blackwell Publishing.

[pce70714-bib-0068] Riederer, M. , and G. Schneider . 1990. “The Effect of the Environment on the Permeability and Composition of Citrus Leaf Cuticles.” Planta 180: 154–165. 10.1007/BF00193990. 24201939

[pce70714-bib-0069] Riederer, M. , and L. Schreiber . 2001. “Protecting Against Water Loss: Analysis of the Barrier Properties of Plant Cuticles.” Journal of Experimental Botany 52, no. 363: 2023–2032. 10.1093/jexbot/52.363.2023. 11559738

[pce70714-bib-0071] Sadler, C. , B. Schroll , V. Zeisler , F. Waßmann , R. Franke , and L. Schreiber . 2016. “Wax and Cutin Mutants of Arabidopsis: Quantitative Characterization of the Cuticular Transport Barrier in Relation to Chemical Composition.” Biochimica et Biophysica Acta (BBA)—Molecular and Cell Biology of Lipids 1861, no. 9 pt. B: 1336–1344. 10.1016/j.bbalip.2016.03.002. 26965486

[pce70714-bib-0072] Samuels, L. , L. Kunst , and R. Jetter . 2008. “Sealing Plant Surfaces: Cuticular Wax Formation by Epidermal Cells.” Annual Review of Plant Biology 59: 683–707. 10.1146/annurev.arplant.59.103006.093219. 18251711

[pce70714-bib-0073] Santamaria, J. M. , and G. Kerstiens . 1994. “The Lack of Control of Water Loss in Micropropagated Plants Is Not Related to Poor Cuticle Development.” Physiologia Plantarum 91, no. 2: 191–195. 10.1111/j.1399-3054.1994.tb00418.x.

[pce70714-bib-0074] Schlegel, T. K. , J. Schönherr , and L. Schreiber . 2005. “Size Selectivity of Aqueous Pores in Stomatous Cuticles of *Vicia faba* Leaves.” Planta 221, no. 5: 648–655. 10.1007/s00425-005-1480-1. 15700185

[pce70714-bib-0075] Schönherr, J. 2000. “Calcium Chloride Penetrates Plant Cuticles via Aqueous Pores.” Planta 212: 112–118. 10.1007/s004250000373. 11219575

[pce70714-bib-0076] Schonherr, J. 2006. “Characterization of Aqueous Pores in Plant Cuticles and Permeation of Ionic Solutes.” Journal of Experimental Botany 57, no. 11: 2471–2491. 10.1093/jxb/erj217. 16825315

[pce70714-bib-0077] Schönherr, J. , and K. Lendzian . 1981. “A Simple and Inexpensive Method of Measuring Water Permeability of Isolated Plant Cuticular Membranes.” Zeitschrift für Pflanzenphysiologie 102, no. 4: 321–327. 10.1016/S0044-328X(81)80203-6.

[pce70714-bib-0085] Schönherr, J. , and L. Schreiber . 2004. “Size Selectivity of Aqueous Pores in Astomatous Cuticular Membranes Isolated From *Populus canescens* (Aiton) Sm. Leaves.” Planta 219, no. 3: 405–411. 10.1007/s00425-004-1239-0. 15024648

[pce70714-bib-0078] Schönherr, J. , and M. Riederer . 1989. “Foliar Penetration and Accumulation of Organic Chemicals in Plant Cuticles. Vol. 108.” In Reviews of Environmental Contamination and Toxicology, edited by G. W. Ware . Springer.

[pce70714-bib-0080] Schreiber, L. 2006. “Characterisation of Polar Paths of Transport in Plant Cuticles.” In Annual Plant Reviews Vol. 23: Biology of the Plant Cuticle, edited by M. Riederer and C. Müller , Blackwell Publishing, 280–291.

[pce70714-bib-0083] Schreiber, L. , and J. Schönherr . 2009. Water and Solute Permeability of Plant Cuticles: Measurement and Data Analysis. Springer.

[pce70714-bib-0081] Schreiber, L. , and M. Riederer . 1996a. “Determination of Diffusion Coefficients of Octadecanoic Acid in Isolated Cuticular Waxes and Their Relationship to Cuticular Water Permeabilities.” Plant, Cell & Environment 19, no. 9: 1075–1082. 10.1111/j.1365-3040.1996.tb00214.x.

[pce70714-bib-0082] Schreiber, L. , and M. Riederer . 1996b. “Ecophysiology of Cuticular Transpiration: Comparative Investigation of Cuticular Water Permeability of Plant Species From Different Habitats.” Oecologia 107: 426–432. 10.1007/BF00333931. 28307383

[pce70714-bib-0079] Schreiber, L. , S. Elshatshat , K. Koch , J. Lin , and J. Santrucek . 2006. “AgCl Precipitates in Isolated Cuticular Membranes Reduce Rates of Cuticular Transpiration.” Planta 223, no. 2: 283–290. 10.1007/s00425-005-0084-0. 16151845

[pce70714-bib-0084] Schuster, A.‐C. , M. Burghardt , and M. Riederer . 2017. “The Ecophysiology of Leaf Cuticular Transpiration: Are Cuticular Water Permeabilities Adapted to Ecological Conditions?.” Journal of Experimental Botany 68, no. 19: 5271–5279. 10.1093/jxb/erx321. 29036342

[pce70714-bib-0087] Seo, P. J. , and C. M. Park . 2011. “Cuticular Wax Biosynthesis as a Way of Inducing Drought Resistance.” Plant Signaling & Behavior 6, no. 7: 1043–1045. 10.4161/psb.6.7.15606. PMC3257791 21617381

[pce70714-bib-0086] Seo, P. J. , S. B. Lee , M. C. Suh , M. J. Park , Y. S. Go , and C. M. Park . 2011. “The MYB96 Transcription Factor Regulates Cuticular Wax Biosynthesis Under Drought Conditions in Arabidopsis.” Plant Cell 23, no. 3: 1138–1152. 10.1105/tpc.111.083485. PMC3082259 21398568

[pce70714-bib-0088] Seufert, P. , S. Staiger , K. Arand , A. Bueno , M. Burghardt , and M. Riederer . 2022. “Building a Barrier: The Influence of Different Wax Fractions on the Water Transpiration Barrier of Leaf Cuticles.” Frontiers in Plant Science 12: 766602. 10.3389/fpls.2021.766602. PMC8766326 35069622

[pce70714-bib-0089] Shaheenuzzamn, M. , S. Shi , K. Sohail , et al. 2021. “Regulation of Cuticular Wax Biosynthesis in Plants Under Abiotic Stress.” Plant Biotechnology Reports 15, no. 1: 1–12. 10.1007/s11816-020-00656-z.

[pce70714-bib-0090] Shellakkutti, N. , P. D. Thangamani , K. Suresh , et al. 2022. “Cuticular Transpiration Is Not Affected by Enhanced Wax and Cutin Amounts in Response to Osmotic Stress in Barley.” Physiologia Plantarum 174, no. 4: e13735. 10.1111/ppl.13735. 35716005

[pce70714-bib-0091] Shepherd, T. , and D. Wynne Griffiths . 2006. “The Effects of Stress on Plant Cuticular Waxes.” New Phytologist 171, no. 3: 469–499. 10.1111/j.1469-8137.2006.01826.x. 16866954

[pce70714-bib-0092] Steinmüller, D. , and M. Tevini . 1985. “Action of Ultraviolet Radiation (UV‐B) Upon Cuticular Waxes in Some Crop Plants.” Planta 164: 557–564. 10.1007/BF00395975. 24248232

[pce70714-bib-0093] Sumbur, B. , M. Zhou , T. Dorjee , et al. 2024. “Chemical and Transcriptomic Analyses of Leaf Cuticular Wax Metabolism in *Ammopiptanthus mongolicus* Under Osmotic Stress.” Biomolecules 14, no. 2: 227. 10.3390/biom14020227. PMC10886927 38397464

[pce70714-bib-0094] Sutter, E. , and R. W. Langhans . 1982. “Formation of Epicuticular Wax and Its Effect on Water Loss in Cabbage Plants Regenerated From Shoot‐Tip Culture.” Canadian Journal of Botany 60, no. 12: 2896–2902. 10.1139/b82-350.

[pce70714-bib-0095] Taiz, L. and Zeiger, E. 2003. *Plant Physiology*. 3rd ed. Vol. 91. Sinauer Associates.

[pce70714-bib-0096] Tiloca, G. , O. Buchner , M. Stegner , N. Gierlinger , and G. Neuner . 2026. “Phenotypic Cuticle Plasticity at High Elevation: Is Microstructure and Microchemistry Related to Water Permeability?.” Plant, Cell & Environment 49: 1778–1792. 10.1111/pce.70344. PMC12873512 41413967

[pce70714-bib-0107] Vogg, G. , S. Fischer , J. Leide , et al. 2004. “Tomato Fruit Cuticular Waxes and Their Effects on Transpiration Barrier Properties: Functional Characterization of a Mutant Deficient in a Very‐Long‐Chain Fatty Acid ‐Ketoacyl‐CoA Synthase.” Journal of Experimental Botany 55, no. 401: 1401–1410. 10.1093/jxb/erh149. 15133057

[pce70714-bib-0097] Whitecross, M. , and D. Armstrong . 1972. “Environmental Effects on Epicuticular Waxes of *Brassica napus* L.” Australian Journal of Botany 20, no. 1: 87–95. 10.1071/BT9720087.

[pce70714-bib-0098] Whittaker, R. H. , and G. E. Likens . 1975. “The Biosphere and Man.” In Primary Productivity of the Biosphere, edited by H. Lieth and R. H. Whittaker , 14, 305–328. Springer. 10.1007/978-3-642-80913-2_15.

[pce70714-bib-0100] Yang, F. , Y. Han , Q.‐H. Zhu , et al. 2022. “Impact of Water Deficiency on Leaf Cuticle Lipids and Gene Expression Networks in Cotton (*Gossypium hirsutum* L.).” BMC Plant Biology 22, no. 1: 404. 10.1186/s12870-022-03788-2. PMC9382817 35978290

[pce70714-bib-0101] Yeats, T. H. , and J. K. C. Rose . 2013. “The Formation and Function of Plant Cuticles.” Plant Physiology 163, no. 1: 5–20. 10.1104/pp.113.222737. PMC3762664 23893170

[pce70714-bib-0103] Zeisler‐Diehl, V. , Y. Müller , and L. Schreiber . 2018. “Epicuticular Wax on Leaf Cuticles Does Not Establish the Transpiration Barrier, Which Is Essentially Formed by Intracuticular Wax.” Journal of Plant Physiology 227: 66–74. 10.1016/j.jplph.2018.03.018. 29653782

[pce70714-bib-0102] Zeisler, V. , and L. Schreiber . 2016. “Epicuticular Wax on Cherry Laurel (*Prunus laurocerasus*) Leaves Does Not Constitute the Cuticular Transpiration Barrier.” Planta 243, no. 1: 65–81. 10.1007/s00425-015-2397-y. PMC4698295 26341347

[pce70714-bib-0104] Zhang, P. , Y. Zou , B. Song , et al. 2022. “Cuticular Lipids and Associated Gene Expression Analysis Under NaCl Stress in *Thellungiella salsuginea* .” Physiologia Plantarum 174, no. 1: e13625. 10.1111/ppl.13625. 35023161

[pce70714-bib-0105] Zhang, S. , S. Wu , Q. Hou , et al. 2024. “Fatty Acid De Novo Biosynthesis in Plastids: Key Enzymes and Their Critical Roles for Male Reproduction and Other Processes in Plants.” Plant Physiology and Biochemistry 210: 108654. 10.1016/j.plaphy.2024.108654. 38663264

[pce70714-bib-0106] Zhang, Y. , Z. Du , Y. Han , et al. 2021. “Plasticity of the Cuticular Transpiration Barrier in Response to Water Shortage and Resupply in *Camellia sinensis*: A Role of Cuticular Waxes.” Frontiers in Plant Science 11: 600069. 10.3389/fpls.2020.600069. PMC7829210 33505410

