# Supplementary material for: Cuticular Waxes in Altered Environments and Their Role in Leaf Water Loss Dynamics
Source: Plant Cell Environ. 2026 Jul 6;49(11):8014–33. doi: 10.1111/pce.70714 (PMC13646681; doi:10.1111/pce.70714)
Supplement: Supplementary file 1 [file PCE-49-8014-s001.docx]

# S2 Literature selection

Considering the abundance of research investigating the relationships between environmental conditions and/or cuticular waxes and/or residual (cuticular) transpiration (109 publications were analyzed), strict inclusion criteria for available publications had to be defined in order to enable and standardize comparisons between formerly independent studies. In this context, it should briefly be noted that the majority of selected data is based on highly bred model species, which have been genetically altered during domestication or even by targeted genetic modifications, and thus cannot entirely capture the existing biological and ecological diversity. Nonetheless, the filtered studies still cover a diverse range of ecologically and economically important species that provide a robust foundation for our reassessment. Apart from a generally hypothesis-driven study layout (effect of environment **→** wax vs. effect of wax **→** transpiration), great emphasis was placed on the utilization of state-of-the-art methodologies of chemical analysis and/or residual (cuticular) transpiration measurements. All studies that were screened considering the established inclusion criteria and ultimately either included (31 publications) or rejected (78 publications) are listed in Table S1.

## S2.1 Interspecific variation

A common approach to link cuticular wax with residual (cuticular) transpiration, especially in older research, is to compare species that naturally differ in both traits (for example, comparing ‘high wax’ to ‘low wax’ species). While such comparisons appear intuitive, they neglect fundamental interspecific differences in cuticular features beyond wax quantity as potential confounding variables, including (but not limited to) the cuticular ultrastructure, cutin organization, and wax composition. As a result, a direct lineup of independent species rather represents a ‘comparing apples to oranges’ scenario in which interspecific dissimilarities can lead to false assumptions of causal relationships. Comparisons of cultivars are often regarded as acceptable given that they generally share a closer genetic background, but already minor differences in gene expression (for instance, resulting from random mutations and/or adaptations to different ecological niches) could still confound the results to a certain degree. Although the impact of such intraspecific factors is certainly open for debate, the restriction of the inclusion of studies to within-cultivar comparisons in this review ensures the most robust assessment of how altered cuticular wax can affect residual (cuticular) transpiration in leaves.

## S2.2 Leaves as research emphasis

This review will focus on leaves (excluding data from fruits, flowers, or stems), given that they represent the most informative organs for studying the cuticle’s role in water regulation. To approximate *in vivo* conditions, we further concentrated on studies that determined cuticular wax loads and water loss rates from whole leaves rather than on data obtained from enzymatically isolated leaf cuticles. Although this chosen approach can never fully exclude the potentially confounding factor of partially open stomata (as will be discussed again in Subsection 4.3), it can, at least in a variety of species (Kerstiens 1996; Burghardt and Riederer 2003; Burghardt et al. 2008; Grünhofer et al. 2022) but certainly not all (Burghardt and Riederer 2003; Kerstiens 1996; Duursma et al. 2019), effectively be accounted for by methodologically identifying and excluding the stomatal contribution (Subsection S2.4).

## S2.3 Cuticular wax analysis

For cuticular wax analysis, this review focused on research that employed gas chromatography rather than solely relying on gravimetric measurements of organic solvent extracts. This technique not only allows for a more precise quantification of cuticular wax amounts but also enables investigations of the qualitative cuticular wax composition (Tables 1-3). Because gravimetric methods dominate much of the older research but lack this high-quality resolution, these studies were separately compiled in Table S2. Care was taken that the included studies related the absolute cuticular wax amount to the extracted leaf surface area (µg cm^−2^) instead of leaf fresh or dry mass. Normalization by leaf weight can obscure the true concentration of cuticular wax on the leaf when leaf weights change independently (e.g., as a result of varying leaf thickness, water content, or lignification), making ‘heavier’ leaves appear to have less wax compared to ‘lighter’ leaves. In Tables 1-3, the reported changes in the absolute cuticular wax amount or relative cuticular wax composition reflect the conclusions drawn by the authors of the respective publication. However, these interpretations can be shaped by subjective judgment and do not always allow for straightforward comparisons across studies (for example, what is interpreted as an effect of high relevance by one author is not necessarily considered meaningful by another). Therefore, applying standardized thresholds to categorize a response as ‘meaningful’ is essential to enable reliable comparisons between different studies. To address this, we defined uniform threshold values for changes in the absolute cuticular wax amount and its relative composition. Changes in total wax amounts were categorized as ‘meaningful’ if they exceeded >1.25–fold or <0.75–fold (±25% of the control treatment wax amount), as we presume that smaller variations might still fall within underlying natural variation. In the case of wax composition, defining universal thresholds is more complicated, given that cuticular wax profiles vary greatly between species. To still allow for a coherent comparison across species and studies, we focused on only the relative abundance of major aliphatic compound classes (acids, alcohols, alkanes, aldehydes, ketones, and esters). To distinguish substantial shifts from minor (natural) fluctuations, we defined a threshold of ±10 percentage points (pp) in the relative abundance of one or more compound classes as indicative of a ‘meaningful’ physiological response. In cases where our reassessment differs from the conclusion drawn in the original publication, parentheses ([…]) were added in the ‘Effect on wax amount/composition’ (Table 1) or ‘Change in wax amount/composition’ (Tables 2 and 3) columns while the truly reported values were stated in the ‘Absolute change’ or ‘Relative change’ columns.

## S2.4 Quantifying cuticular water barrier properties

Especially the estimation and contextualization of cuticular water permeability is difficult to compare, given that there are a lot of different methods to allegedly do so. In the following segments, we will critically examine common approaches for assessing cuticular water loss, highlight potential pitfalls, and culminate in what we consider the most robust strategy to quantify residual (cuticular) transpiration.

### S2.4.1 Oversimplification of drought resistance

In some studies, conclusions about cuticular water permeability as a contributor to the overall drought resistance are based just on visual indicators such as delayed wilting or improved plant performance after water withdrawal – proxies that are highly indirect and influenced by numerous confounding factors unrelated to the cuticle, including (but not limited to) the regulation of stomatal closure, osmotic adjustment through solute accumulation, or broader anatomical modifications such as an optimization of the leaf area-to-volume ratio. Because visual assessment reflects a collection of whole-plant responses, it does not allow clear conclusions about the contribution of cuticular permeability as a single factor and should be interpreted with great caution. Studies relying on this approach were therefore excluded from this review. Seemingly internalizing this logic, more targeted investigations of the cuticular barrier properties are frequently assessed by chlorophyll leaching assays and gravimetric transpiration measurements.

### S2.4.2 Chlorophyll leaching assays

However, particularly chlorophyll leaching assays pose fundamental conceptual limitations when used trying to quantify the cuticle’s function as a water barrier. Typically, excised leaves are immersed in 80% ethanol for several hours and the release of large chlorophyll molecules into the solvent is monitored over time. The assay postulates that chlorophyll diffuses through the intact cuticle and dissolves in the outer 80% ethanol solution, and observed differences in chlorophyll release into the liquid medium (for instance, between different genotypes or in response to environmental stress) are attributed to changes in the cuticular barrier. Such leaching kinetics are then often arbitrarily paralleled to the diffusion kinetics of water molecules while intact leaves are being exposed to the gas phase of the atmosphere. This approach is highly questionable in the opinion of the authors of this review, due to several accumulating reasons: (i) The primary barrier to water movement across the cuticle undeniably resides within the wax layer, which itself is likely, at least partially, solubilized in 80% ethanol (Holser 2009; Castellanos and Rojas‐Pérez 2024). In other words, the assay gradually removes the very barrier it aims to evaluate. Considering this, it is not surprising that in wax-deficient mutants, where the cuticular wax layer is already compromised and thus dissolves faster, a more rapid onset of chlorophyll leakage is observed. Conversely, increased wax loads result in delayed tissue penetration because it takes longer to dissolve the functional wax barrier. (ii) Beyond this early stage of chlorophyll release, the slopes of the chlorophyll extraction kinetics tend to equalize across different genotypes or treatments – a similarity that is often overlooked. It appears that once the cuticular barrier is compromised, rates of chlorophyll diffusion are independent of the initial inhibitory wax amounts. Given that the plasma membrane and the chloroplast envelope need to be disrupted to solubilize chlorophyll, it is highly unlikely that the cuticle, as the outermost layer in direct contact with the organic solvent, remains unaffected and intact. Once the cuticular wax and membrane barriers are dissolved, a constant efflux of chlorophyll into the solvent is guaranteed. (iii) Due to its large molar mass (893 g mol⁻¹), chlorophyll should not be able to cross an intact cuticle by simple diffusion within the usual measurement period of only a few hours (based on penetration studies with Populus × canescens and *Vicia faba* leaf cuticles, a threshold of around 400-500 g mol⁻¹ can be expected; Schönherr and Schreiber 2004; Schlegel et al. 2005). This further underscores that the assay likely reflects chlorophyll leaching out of damaged tissue and infiltrated stomata rather than truly quantifying unaltered cuticular permeability. (iv) Differences in stomatal density or efficiency of stomatal closure between genotypes or treatments are rarely considered, yet these variables may substantially affect infiltration kinetics and severely confound the results when submerging typically hypo- or amphistomatous leaves in organic solvent. Considering all these factors, chlorophyll leaching assays are not an appropriate approach to assess the cuticle’s function as a water barrier at all. Rather, they integrate multiple processes, including the rate of wax solubilization, the breakdown of cellular and plastid membranes, and potential differences in stomatal infiltration. Thus, this method at best provides a rough proxy for the efficiency of solvent penetration into cells and plastids (which might be slowed down by a thicker wax layer in the very beginning), but at worst can establish a misleading perception of the cuticle’s role in water regulation. Studies that based their assessment of cuticular water permeability on chlorophyll leaching assays were excluded from this review.

### S2.4.3 Gravimetric transpiration measurements

In contrast, gravimetric transpiration measurements are well established and generally regarded as a reliably standardized method*.* By monitoring the reduction in leaf weight of detached but otherwise intact (hereafter referred to as ‘whole’) leaves, the cumulative evaporation-driven weight loss over time can be used to quantify the cuticle’s water barrier properties. While this method is experimentally straightforward, there are some pitfalls primarily in data evaluation that need to be carefully considered: (i) residual transpiration kinetics are frequently expressed as relative weight loss, i.e., the weight of water lost over time is related to the initial leaf fresh weight. Those two variables, however, are not independent from one another: For instance, if genetic manipulation or environmental stress increases the leaf dry weight (e.g., as a result of promoted lignification) the relative water loss (% of leaf fresh weight) can appear lower even when the actual amount of water lost is the same, thus substantially obscuring the true results. (ii) Leaf surface areas are often not considered in this approach, although normalization to leaf area is essential to accurately reflect differences in residual (cuticular) transpiration, especially when comparing leaves of different sizes and surface-to-volume ratios. Ignoring surface area can make smaller leaves appear to lose less water than larger ones, even when the transpiration rates per unit surface area are identical. Finally, (iii) stomatal closure shortly after leaf detachment necessarily needs to be considered when working with whole leaves, but is nonetheless recurrently neglected, possibly resulting in a substantial overestimation of the true cuticular transpiration. A reliable method to identify and exclude the bulk of the initial stomatal contribution is by plotting incremental weight loss per leaf surface area (the slope between two consecutive measurements) against the corresponding relative water deficit (the incremental weight loss related to the leaf fresh weight) and visually selecting values that lie within the plateauing region of the resulting graph. While incremental weight loss is typically elevated during the initial phase of the assay due to partially still open stomata, the plateau represents the residual (cuticular) transpiration (Schuster et al. 2016).

### S2.4.4 Establishing comparability by a standardized metric

Accounting for the limitations outlined above, a more appropriate and physiologically meaningful measure of cuticular water loss is to estimate the permeance (Zeisler-Diehl et al. 2017). The permeance (m s^−1^) can be calculated by dividing the slope of leaf surface area‐normalized water loss over time (g m^−2^ s^−1^) by the driving force, which equals the water vapor concentration gradient between the fully saturated leaf interior and the surrounding atmosphere (assumed to approximate the density of water, 10^6^ g m^−3^ at 25°C). This methodological approach is independent of possible alterations in cuticular integrity or altered leaf dry weights and surface areas and provides a high degree of comparability across different species, genotypes, treatments, and even plant organs (Schuster et al. 2016; Grünhofer and Schreiber 2023). In this review, only studies that incorporated permeances based on such residual (cuticular) transpiration measurements (excluding the initial stomatal contribution) in combination with high-quality chemical wax analysis were included (Tables 2 and 3). The changes in permeance reported in Tables 2 and 3 reflect the conclusions drawn by the authors of the respective study. In analogy to the absolute cuticular wax amount (Subsection S2.3), changes in permeance were categorized as ‘meaningful’ if they exceeded >1.25–fold or <0.75–fold (±25% of the control treatment permeance). Our reassessment did not differ from the conclusion drawn in the original publication in any case.

# Table S1: Complete collection of all 109 studies investigating the relationships between environmental conditions and/or cuticular waxes and/or residual (cuticular) transpiration that were screened for this review, and that were either included (31 studies) or excluded (78 studies) under the established inclusion criteria (Section S2, Subsections S2.1 to S2.4). ×, study was excluded; o, gravimetric data only included in Table S2 but not in the main manuscript; ✓, study was included in the main manuscript.

| **Reference** | **Species** | **Reasoning** | **Excluded/**  **Included** |
| --- | --- | --- | --- |
| Effect of the environment on cuticular wax | | | |
| Bueno et al.(2019b) | *Phoenix dactylifera* vs. *Citrullus colocynthis* | **2.1 No focus on a single species** | × |
| Oliveira et al. (2003) | Comparing 4 species from the Caatinga to 4 species from the Cerrado | **2.1 No focus on a single species** | × |
| Bian et al. (2022) | *Hordeum vulgare* | 2.1 Fulfilled, **2.2 No focus on whole leaves** | × |
| Leide et al. (2007) | *Solanum lycopersicum* | 2.1 Fulfilled, **2.2 No focus on whole leaves** | × |
| Hauke and Schreiber (1998) | *Hedera helix* | 2.1 Fulfilled, **2.2 No focus on whole leaves** | × |
| Romero and Rose (2019) | *Solanum lycopersicum* | 2.1 Fulfilled, **2.2 No focus on whole leaves** | × |
| VanderWeide et al. (2022) | *Vitis vinifera* | 2.1 Fulfilled, **2.2 No focus on whole leaves** | × |
| Vogg et al. (2004) | *Solanum lycopersicum* | 2.1 Fulfilled, **2.2 No focus on whole leaves** | × |
| Xu et al. (2025) | *Xanthoceras sorbifolium* | 2.1 Fulfilled, **2.2 No focus on whole leaves** | × |
| Elham et al. (2012) | *Triticum aestivum* | 2.1 Fulfilled, 2.2 Fulfilled, **2.3 No gas chromatography (GC-FID)** | × |
| Huggins et al. (2017) | *Triticum aestivum* | 2.1 Fulfilled, 2.2 Fulfilled, **2.3 No gas chromatography (GC-FID)** | × |
| DeLucia and Berlyn (1984) | *Abies balsamera* | 2.1 Fulfilled, 2.2 Fulfilled, **2.3 No gas chromatography (GC-FID)** | × |
| Gonzàlez and Ayerbe (2010) | *Hordeum vulgare* | 2.1 Fulfilled, 2.2 Fulfilled, **2.3 No gas chromatography (GC-FID)** | × |
| Hadley and Smith (1990) | *Picea engelmannii, Abies lasiocarpa, Pseudotsuga menziesii, Pinus* spp. | 2.1 Fulfilled, 2.2 Fulfilled, **2.3 No gas chromatography (GC-FID)** | × |
| Premachandra et al. (1992) | *Sorghum bicolor* | 2.1 Fulfilled, 2.2 Fulfilled, **2.3 No gas chromatography (GC-FID)** | × |
| Sanchez et al. (2001) | *Pisum sativum* | 2.1 Fulfilled, 2.2 Fulfilled, **2.3 No gas chromatography (GC-FID)** | × |
| Spoorthi et al. (2025) | *Oryza sativa* | 2.1 Fulfilled, 2.2 Fulfilled, **2.3 No gas chromatography (GC-FID)** | × |
| Abdullah et al. (2021) | *Arabidopsis thaliana, Camelina sativa* | 2.1 Fulfilled, 2.2 Fulfilled, **2.3 No surface area-normalized wax amounts** | × |
| Al-Abdallat et al. (2014) | *Solanum lycopersicum* | 2.1 Fulfilled, 2.2 Fulfilled, **2.3 No surface area-normalized wax amounts** | × |
| Hull (1958) | *Prosopis juliflora* | 2.1 Fulfilled, 2.2 Fulfilled, **2.3 No surface area-normalized wax amounts** | × |
| Jiang et al. (2025) | *Poa pratensis* | 2.1 Fulfilled, 2.2 Fulfilled, **2.3 No surface area-normalized wax amounts** | × |
| Le Provost et al. (2013) | *Pinus pinaster* | 2.1 Fulfilled, 2.2 Fulfilled, **2.3 No surface area-normalized wax amounts** | × |
| Luo et al. (2013) | *Arabidopsis thaliana* | 2.1 Fulfilled, 2.2 Fulfilled, **2.3 No surface area-normalized wax amounts** | × |
| Qin et al. (2011) | *Oryza sativa* | 2.1 Fulfilled, 2.2 Fulfilled, **2.3 No surface area-normalized wax amounts** | × |
| Pilon et al. (1999) | *Poa* spp. | 2.1 Fulfilled, 2.2 Fulfilled, **2.3 No surface area-normalized wax amounts** | × |
| Yang et al.(2011a) | *Arabidopsis thaliana* | 2.1 Fulfilled, 2.2 Fulfilled, **2.3 No surface area-normalized wax amounts** | × |
| Zhu et al. (2014) | *Arabidopsis thaliana* | 2.1 Fulfilled, 2.2 Fulfilled, **2.3 No surface area-normalized wax amounts** | × |
| Barnes (1996) | Nicotiana tabacum | 2.1 Fulfilled, 2.2 Fulfilled, **2.3 Gravimetric measurement of organic solvent extracts** | o, Table S2 |
| Bengston et al. (1978) | *Avena sativa* | 2.1 Fulfilled, 2.2 Fulfilled, **2.3 Gravimetric measurement of organic solvent extracts** | o, Table S2 |
| Bondada et al. (1996) | *Gossypium hirsutum* | 2.1 Fulfilled, 2.2 Fulfilled, **2.3 Gravimetric measurement of organic solvent extracts** | o, Table S2 |
| Darnell and Ferreen(1983) | *Malus domestica* | 2.1 Fulfilled, 2.2 Fulfilled, **2.3 Gravimetric measurement of organic solvent extracts** | o, Table S2 |
| Gauvrit and Gaillardon (1991) | *Zea mays* | 2.1 Fulfilled, 2.2 Fulfilled, **2.3 Gravimetric measurement of organic solvent extracts** | o, Table S2 |
| Giese (1975) | *Hordeum vulgare* | 2.1 Fulfilled, 2.2 Fulfilled, **2.3 Gravimetric measurement of organic solvent extracts** | o, Table S2 |
| Gonzalez et al. (1996) | Pisum sativum | 2.1 Fulfilled, 2.2 Fulfilled, **2.3 Gravimetric measurement of organic solvent extracts** | o, Table S2 |
| Jefferson et al. (1989) | Medicago sativa, *Agropyron desertorum* | 2.1 Fulfilled, 2.2 Fulfilled, **2.3 Gravimetric measurement of organic solvent extracts** | o, Table S2 |
| Kakani et al. (2003) | *Gossypium hirsutum* | 2.1 Fulfilled, 2.2 Fulfilled, **2.3 Gravimetric measurement of organic solvent extracts** | o, Table S2 |
| Letchamo and Gosselin (1996) | *Thymus vulgaris* | 2.1 Fulfilled, 2.2 Fulfilled, **2.3 Gravimetric measurement of organic solvent extracts** | o, Table S2 |
| Qaderi and Reid (2005) | *Brassica napus* | 2.1 Fulfilled, 2.2 Fulfilled, **2.3 Gravimetric measurement of organic solvent extracts** | o, Table S2 |
| Reed and Tukey (1982) | *Brassica oleracea, Dianthus caryophyllus* | 2.1 Fulfilled, 2.2 Fulfilled, **2.3 Gravimetric measurement of organic solvent extracts** | o, Table S2 |
| Sutter and Langhans (1982) | *Brassica oleracea* | 2.1 Fulfilled, 2.2 Fulfilled, **2.3 Gravimetric measurement of organic solvent extracts** | o, Table S2 |
| Whitecross and Armstrong (1972) | *Brassica napus* | 2.1 Fulfilled, 2.2 Fulfilled, **2.3 Gravimetric measurement of organic solvent extracts** | o, Table S2 |
| Baker (1974) | *Brassica oleracea* | 2.1 Fulfilled, 2.2 Fulfilled, 2.3 Fulfilled | ✓, Table 1 |
| Cameron et al. (2006) | *Nicotiana glauca* | 2.1 Fulfilled, 2.2 Fulfilled, 2.3 Fulfilled | ✓, Table 1 |
| Chen et al. (2020) | *Cammelia sinensis* | 2.1 Fulfilled, 2.2 Fulfilled, 2.3 Fulfilled | ✓, Table 1 |
| De Araujo Silva et al. (2021) | *Ricinus communis* | 2.1 Fulfilled, 2.2 Fulfilled, 2.3 Fulfilled | ✓, Table 1 |
| Even et al. (2018) | *Hordeum vulgare* | 2.1 Fulfilled, 2.2 Fulfilled, 2.3 Fulfilled | ✓, Table 1 |
| Fricke et al.(2006) | *Hordeum vulgare* | 2.1 Fulfilled, 2.2 Fulfilled, 2.3 Fulfilled | ✓, Table 1 |
| Go et al. (2014) | *Arabidopsis thaliana* | 2.1 Fulfilled, 2.2 Fulfilled, 2.3 Fulfilled | ✓, Table 1 |
| Grünhofer et al.(2025) | *Hordeum vulgare* | 2.1 Fulfilled, 2.2 Fulfilled, 2.3 Fulfilled | ✓, Table 1 |
| He et al. (2019) | *Thellungiella salsuginea* | 2.1 Fulfilled, 2.2 Fulfilled, 2.3 Fulfilled | ✓, Table 1 |
| Jenks et al. (2001) | *Rosa* × *hybrida* | 2.1 Fulfilled, 2.2 Fulfilled, 2.3 Fulfilled | ✓, Table 1 |
| Kim et al. (2007a) | *Glycine max* | 2.1 Fulfilled, 2.2 Fulfilled, 2.3 Fulfilled | ✓, Table 1 |
| Kim et al. (2007b) | *Sesamum indicum* | 2.1 Fulfilled, 2.2 Fulfilled, 2.3 Fulfilled | ✓, Table 1 |
| Koch et al. (2006) | *Brassica oleracea, Eucalyptus gunnii, Tropaeolum majus* | 2.1 Fulfilled, 2.2 Fulfilled, 2.3 Fulfilled | ✓, Table 1 |
| Kosma et al. (2009) | *Arabidopsis thaliana* | 2.1 Fulfilled, 2.2 Fulfilled, 2.3 Fulfilled | ✓, Table 1 |
| Lykholat et al. (2020) | *Ulmus pumila, Ulmus minor* | 2.1 Fulfilled, 2.2 Fulfilled, 2.3 Fulfilled | ✓, Table 1 |
| Ni et al. (2012) | *Medicago sativa* | 2.1 Fulfilled, 2.2 Fulfilled, 2.3 Fulfilled | ✓, Table 1 |
| Patwari et al. (2019) | *Arabidopsis thaliana* | 2.1 Fulfilled, 2.2 Fulfilled, 2.3 Fulfilled | ✓, Table 1 |
| Rahman et al. (2021) | *Arabidopsis thaliana* | 2.1 Fulfilled, 2.2 Fulfilled, 2.3 Fulfilled | ✓, Table 1 |
| Steinmüller and Tevini (1985) | *Hordeum vulgare,* Phaseolus vulgaris | 2.1 Fulfilled, 2.2 Fulfilled, 2.3 Fulfilled | ✓, Table 1 |
| Sumbur et al. (2024) | *Ammopiptanthus mongolicus* | 2.1 Fulfilled, 2.2 Fulfilled, 2.3 Fulfilled | ✓, Table 1 |
| Yang et al. (2022) | *Gossypium hirsutum* | 2.1 Fulfilled, 2.2 Fulfilled, 2.3 Fulfilled | ✓, Table 1 |
| Zhang et al. (2021) | *Camellia sinensis* | 2.1 Fulfilled, 2.2 Fulfilled, 2.3 Fulfilled | ✓, Table 1 |
| Zhang et al. (2022) | *Thellungiella salsuginea* | 2.1 Fulfilled, 2.2 Fulfilled, 2.3 Fulfilled | ✓, Table 1 |
| Herzig et al. (2025) | *Hordeum vulgare, Zea mays, Solanum lycopersicum, Vigna radiata* | 2.1 Fulfilled, 2.2 Fulfilled, 2.3 Fulfilled | ✓, Tables 1, 2 |
| Shellakkutti et al. (2022) | *Hordeum vulgare* | 2.1 Fulfilled, 2.2 Fulfilled, 2.3 Fulfilled | ✓, Tables 1, 2 |
| Effect of cuticular wax on residual (cuticular) transpiration | | | |
| Jetter and Riederer (2016) | Comparing 4 different species | **2.1 No focus on a single species** | × |
| Schreiber and Riederer (1996) | Comparing 24 different species | **2.1 No focus on a single species** | × |
| Seufert et al. (2022) | Comparing 7 different species | **2.1 No focus on a single species** | × |
| Jenks et al. (1994) | *Sorghum bicolor* | 2.1 Fulfilled, 2.2 Fulfilled, **2.3 No gas chromatography (GC-FID)** | × |
| Jordan et al. (1984) | *Sorghum bicolor* | 2.1 Fulfilled, 2.2 Fulfilled, **2.3 No gas chromatography (GC-FID)** | × |
| Hunsche and Noga (2011) | *Prunus avium* | 2.1 Fulfilled, 2.2 Fulfilled, **2.3 No gas chromatography (GC-FID)** | × |
| Zhao et al. (2025) | *Arabidopsis thaliana* | 2.1 Fulfilled, 2.2 Fulfilled, **2.3 No gas chromatography (GC-FID)** | × |
| Simões et al. (2020) | *Quercus suber* | 2.1 Fulfilled, 2.2 Fulfilled, **2.3 No gas chromatography (GC-FID)** | × |
| Luo et al. (2024) | *Arabidopsis thaliana* | 2.1 Fulfilled, 2.2 Fulfilled, **2.3 No surface area-normalized wax amounts** | × |
| Aharoni et al. (2004) | *Arabidopsis thaliana* | 2.1 Fulfilled, 2.2 Fulfilled, 2.3 Fulfilled, **2.4 No water permeance estimation** | × |
| Chen et al. (2021) | *Camellia sinensis* | 2.1 Fulfilled, 2.2 Fulfilled, 2.3 Fulfilled, **2.4 No water permeance estimation** | × |
| Bao et al. (2017) | *Arabidopsis thaliana* | 2.1 Fulfilled, 2.2 Fulfilled, 2.3 Fulfilled, **2.4 No water permeance estimation** | × |
| Bi et al. (2017) | *Triticum aestivum* | 2.1 Fulfilled, 2.2 Fulfilled, 2.3 Fulfilled, **2.4 No water permeance estimation** | × |
| Bi et al. (2018) | *Triticum aestivum* | 2.1 Fulfilled, 2.2 Fulfilled, 2.3 Fulfilled, **2.4 No water permeance estimation** | × |
| Bourdenx et al. (2011) | *Arabidopsis thaliana* | 2.1 Fulfilled, 2.2 Fulfilled, 2.3 Fulfilled, **2.4 No water permeance estimation** | × |
| Chen et al. (2003) | *Arabidopsis thaliana* | 2.1 Fulfilled, 2.2 Fulfilled, 2.3 Fulfilled, **2.4 No water permeance estimation** | × |
| He et al. (2022) | *Triticum aestivum* | 2.1 Fulfilled, 2.2 Fulfilled, 2.3 Fulfilled, **2.4 No water permeance estimation** | × |
| Islam et al.(2009) | *Oryza sativa* | 2.1 Fulfilled, 2.2 Fulfilled, 2.3 Fulfilled, **2.4 No water permeance estimation** | × |
| Kim et al. (2018) | *Arabidopsis thaliana* | 2.1 Fulfilled, 2.2 Fulfilled, 2.3 Fulfilled, **2.4 No water permeance estimation** | × |
| Larsson and Svenningsson (1986) | *Hordeum vulgare* | 2.1 Fulfilled, 2.2 Fulfilled, 2.3 Fulfilled, **2.4 No water permeance estimation** | × |
| Lee et al. (2014) | *Camelia sativa* | 2.1 Fulfilled, 2.2 Fulfilled, 2.3 Fulfilled, **2.4 No water permeance estimation** | × |
| Lokesh et al. (2019) | *Arachis hypogaea* | 2.1 Fulfilled, 2.2 Fulfilled, 2.3 Fulfilled, **2.4 No water permeance estimation** | × |
| Mao et al. (2012) | *Oryza sativa* | 2.1 Fulfilled, 2.2 Fulfilled, 2.3 Fulfilled, **2.4 No water permeance estimation** | × |
| Norris and Bukovac (1972) | *Pyrus communis* | 2.1 Fulfilled, 2.2 Fulfilled, 2.3 Fulfilled, **2.4 No water permeance estimation** | × |
| O’Toole et al. (1979) | *Oryza sativa* | 2.1 Fulfilled, 2.2 Fulfilled, 2.3 Fulfilled, **2.4 No water permeance estimation** | × |
| Seo et al. (2011) | *Arabidopsis thaliana* | 2.1 Fulfilled, 2.2 Fulfilled, 2.3 Fulfilled, **2.4 No water permeance estimation** | × |
| Seo and Park (2011) | *Arabidopsis thaliana* | 2.1 Fulfilled, 2.2 Fulfilled, 2.3 Fulfilled, **2.4 No water permeance estimation** | × |
| Wang et al. (2012) | *Oryza sativa* | 2.1 Fulfilled, 2.2 Fulfilled, 2.3 Fulfilled, **2.4 No water permeance estimation** | × |
| Wang et al. (2015a) | *Cucumis sativus* | 2.1 Fulfilled, 2.2 Fulfilled, 2.3 Fulfilled, **2.4 No water permeance estimation** | × |
| Wang et al. (2015b) | *Cucumis sativus* | 2.1 Fulfilled, 2.2 Fulfilled, 2.3 Fulfilled, **2.4 No water permeance estimation** | × |
| Wang et al. (2018) | *Oryza sativa* | 2.1 Fulfilled, 2.2 Fulfilled, 2.3 Fulfilled, **2.4 No water permeance estimation** | × |
| Yang et al. (2011b) | *Brassica napus* | 2.1 Fulfilled, 2.2 Fulfilled, 2.3 Fulfilled, **2.4 No water permeance estimation** | × |
| Zhang et al. (2005) | *Medicago sativa* | 2.1 Fulfilled, 2.2 Fulfilled, 2.3 Fulfilled, **2.4 No water permeance estimation** | × |
| Zhang et al. (2007) | *Arabidopsis thaliana* | 2.1 Fulfilled, 2.2 Fulfilled, 2.3 Fulfilled, **2.4 No water permeance estimation** | × |
| Zhang et al. (2019) | *Arabidopsis thaliana* | 2.1 Fulfilled, 2.2 Fulfilled, 2.3 Fulfilled, **2.4 No water permeance estimation** | × |
| Zhou et al. (2013a) | *Agrostis stolonifera* | 2.1 Fulfilled, 2.2 Fulfilled, 2.3 Fulfilled, **2.4 No water permeance estimation** | × |
| Zhou et al. (2013b) | *Oryza sativa* | 2.1 Fulfilled, 2.2 Fulfilled, 2.3 Fulfilled, **2.4 No water permeance estimation** | × |
| Bueno et al. (2019a) | *Quercus coccifera* | 2.1 Fulfilled, 2.2 Fulfilled, 2.3 Fulfilled, 2.4 Fulfilled | ✓, Table 2 |
| Grünhofer et al. (2022) | *Populus* × *canescens* | 2.1 Fulfilled, 2.2 Fulfilled, 2.3 Fulfilled, 2.4 Fulfilled | ✓, Table 2 |
| Müller et al. (2023) | *Hordeum vulgare* | 2.1 Fulfilled, 2.2 Fulfilled, 2.3 Fulfilled, 2.4 Fulfilled | ✓, Table 2 |
| Cheng et al. (2023) | *Dianthus caryophyllus* | 2.1 Fulfilled, 2.2 Fulfilled, 2.3 Fulfilled, 2.4 Fulfilled | ✓, Tables 2, 3 |
| Grünhofer et al.(2021) | *Populus* × *canescens, Populus trichocarpa, Populus euphratica* | 2.1 Fulfilled, 2.2 Fulfilled, 2.3 Fulfilled, 2.4 Fulfilled | ✓, Tables 2, 3 |
| Grünhofer et al. (2023) | *Populus* × *canescens* | 2.1 Fulfilled, 2.2 Fulfilled, 2.3 Fulfilled, 2.4 Fulfilled | ✓, Table 3 |

# **Table S2**: Effect of major environmental stimuli on the surface area-normalized absolute cuticular wax amount of leaves. The compiled research is based on gravimetric measurements of organic solvent extracts. Approximate values were estimated from figures when no numerical data was available. Parentheses […] were added in cases where statistical significance was reached in the original study, but the relevance of the response is questioned by the authors of this review: significant changes in the absolute wax amount were categorized as ‘meaningful’ at a threshold of >1.25-fold or <0.75-fold (±25% of the control treatment wax amount). If several cultivars of the same species were tested in parallel within one study (indicated by cvv.), the response was categorized as ‘meaningful’ if it was observed consistently in the majority of cultivars. E, expanding leaves; FD, fully developed leaves; E/FD, mixture of expanding and fully developed leaves; NA, not available

| **Species** | **Leaf state** | **Stimulus** | **Details** | **Effect on wax**  **amount** | **Absolute**  **change** | **Reference** |
| --- | --- | --- | --- | --- | --- | --- |
| *Hordeum vulgare* | E | Light exposure | Darkness vs.  250 µmol m^‑2^ s^‑1^ | ↑ | 2.44-fold | Giese (1975) |
| *Dianthus caryophyllus* | NA | Light exposure | 145 vs. 440 µE m^‑2^ s^‑1^ | ↑ | 1.54-fold at 15°C,  2.47-fold at 25°C | Reed and Tukey (1982) |
| *Thymus vulgaris* (cvv.) | FD | Light exposure | Natural light vs. +200 µmol m^‑2^ s^‑1^ supplementary light | ↑ | 1.33-fold and 1.48-fold  in 2 out of 2 cultivars | Letchamo and Gosselin (1996) |
| *Brassica napus* | FD | Light exposure | 40 vs. 100% off glasshouse light | ↑ | Approx. 1.4-fold at 15°C,  Approx. 4.0-fold at 27°C | Whitecross and Armstrong (1972) |
| *Brassica oleracea* | NA | Light exposure | 145 vs. 440 µE m^‑2^ s^‑1^ | [↑] | 2.22-fold at 15°C,  no change at 25°C | Reed and Tukey (1982) |
| *Brassica napus* | FD | UV-B radiation | 0 vs. 4.2 kJ m^-2^ d^-1^ | ↑ | Approx. 2.0-fold | Qaderi and Reid (2005) |
| *Gossypium hirsutum* | FD | UV-B radiation | 0 vs. 16 kJ m^-2^ d^-1^ | ↑ | Approx. 1.3-fold and 1.8-fold  in ‘squaring’ and ‘flowering’ stage | Kakani et al. (2003) |
| Nicotiana tabacum | FD | UV-B radiation | 0 vs. 5.7 kJ m^-2^ d^-1^ | [=] | 0.59-fold in GR32-3,  no change in the wildtype | Barnes (1996) |
| Pisum sativum (cvv.) | FD | UV-B radiation | 0 vs. 6.5 kJ m^-2^ d^-1^ | [↑] | 1.39-fold  in 1 out of 6 cultivars | Gonzalez et al. (1996) |
| *Brassica oleracea* | NA | Low temperature | 25 vs. 15°C | ↑ | 3.67-fold at 440 µE m^‑2^ s^‑1^,  1.26-fold at 145 µE m^‑2^ s^‑1^ | Reed and Tukey (1982) |
| *Brassica napus* | FD | Low temperature | 21 vs. 15°C | [↑] | Approx. 1.2-fold at 100% light,  Approx. 2.0-fold at 40% light | Whitecross and Armstrong (1972) |
| *Hordeum vulgare* | E | Low temperature | 23 vs. 15°C | ↓ | Approx. 0.7-fold | Giese (1975) |
| *Zea mays* | FD | Low temperature | 22 vs. 17°C | ↓ | 0.71-fold | Gauvrit and Gaillardon (1990) |
| *Dianthus caryophyllus* | NA | Low temperature | 25 vs. 15°C | [↓] | 0.62-fold at 440 µE m^‑2^ s^‑1^,  no change at 145 µE m^‑2^ s^‑1^ | Reed and Tukey (1982) |
| *Brassica napus* | FD | High temperature | 21 vs. 27°C | [↑] | Approx. 1.8-fold at 100% light,  no change at 40% light | Whitecross and Armstrong (1972) |
| *Malus domestica* | NA | High temperature | 20 vs. 25°C | = | No change | Darnell and Ferreen (1983) |
| *Brassica oleracea* | E | Low relative humidity | 100 vs. 35% *in vitro* | ↑ | 4.63-fold | Sutter and Langhans (1982) |
| *Gossypium hirsutum* | FD | Simulated drought | No watering for 2-3 days | ↑ | 1.69-fold | Bondada et al. (1996) |
| *Malus domestica* | NA | Simulated drought | -13 vs. -33 KPa soil water potential | ↑ | 1.39-fold | Darnell and Ferreen (1983) |
| *Agropyron desertorum* | FD | Simulated drought | Irrigated vs. -0.5 MPa | ↑ | Approx. 1.3-fold | Jefferson et al. (1989) |
| *Thymus vulgaris* (cvv.) | FD | Simulated drought | 90 vs. 50% soil water content | ↑ | 1.27-fold and 1.28-fold  in 2 out of 2 cultivars | Letchamo and Gosselin (1996) |
| *Avena sativa* (cvv.) | FD | Simulated drought | Cooling roots for 3 hours to 1°C on  4 consecutive days | [↑] | 1.62-fold  in 1 out of 6 cultivars | Bengston et al. (1978) |
| *Medicago sativa* (cvv.) | FD | Simulated drought | Irrigated vs. -2.35 MPa | [↑] | Approx. 1.1- to 1.3-fold across  3 harvests and 2 cultivars | Jefferson et al. (1989) |

References

**Abdullah, H. M.; Rodriguez, J.; Salacup, J. M.; Castañeda, I. S.; Schnell, D. J.; Pareek, A.; Dhankher, O. P. 2021**: Increased cuticle waxes by overexpression of *WSD1* improves osmotic stress tolerance in *Arabidopsis thaliana* and *Camelina sativa*. In *International Journal of Molecular Sciences* 22 (10). DOI: 10.3390/ijms22105173.

**Aharoni, A.; Dixit, S.; Jetter, R.; Thoenes, E.; van Arkel, G.; Pereira, A. 2004**: The SHINE clade of AP2 domain transcription factors activates wax biosynthesis, alters cuticle properties, and confers drought tolerance when overexpressed in Arabidopsis. In *The Plant Cell* 16 (9), pp. 2463–2480. DOI: 10.1105/tpc.104.022897.

**Al-Abdallat, A. M.; Al-Debei, H. S.; Ayad, J. Y.; Hasan, S. 2014**: Over-expression of SlSHN1 gene improves drought tolerance by increasing cuticular wax accumulation in tomato. In *International Journal of Molecular Sciences* 15 (11), pp. 19499–19515. DOI: 10.3390/ijms151119499.

**Baker, E. A. 1974**: The influence of environment on leaf wax development in *Brassica oleracea* var. *gemmifera*. In *The New Phytologist* 73 (5), pp. 955–966. DOI: 10.1111/j.1469-8137.1974.tb01324.x.

**Bao, S. G.; Shi, J. X.; Luo, F.; Ding, B.; Hao, J. Y.; Xie, X. D.; Sun, S. J. 2017**: Overexpression of *Sorghum* WINL1 gene confers drought tolerance in *Arabidopsis thaliana* through the regulation of cuticular biosynthesis. In *Plant Cell, Tissue and Organ Culture* 128 (2), pp. 347–356. DOI: 10.1007/s11240-016-1114-2.

**Barnes, J. D.; Percy, K. E.; Paul, N. D.; Jones, P.; McLaughlin, C. K.; Mullineaux, P. M. et al. 1996**: The influence of UV-B radiation on the physicochemical nature of tobacco (*Nicotiana tabacum* L.) leaf surfaces. In *Journal of Experimental Botany* 47 (294), pp. 99–109. DOI: 10.1093/jxb/47.1.99.

**Bengston, C.; Larsson, S.; Liljenberg, C. 1978**: Effects of water stress on cuticular transpiration rate and amount and composition of epicuticular wax in seedlings of six oat varieties. In *Physiologia Plantarum* 44 (4), pp. 319–324. DOI: 10.1111/j.1399-3054.1978.tb01630.x.

**Bi, H.; Kovalchuk, N.; Langridge, P.; Tricker, P. J.; Lopato, S.; Borisjuk, N. 2017**: The impact of drought on wheat leaf cuticle properties. In *BMC Plant Biology* 17 (1), p. 85. DOI: 10.1186/s12870-017-1033-3.

**Bi, H.; Shi, J.; Kovalchuk, N.; Luang, S.; Bazanova, N.; Chirkova, L. et al. 2018**: Overexpression of the TaSHN1 transcription factor in bread wheat leads to leaf surface modifications, improved drought tolerance, and no yield penalty under controlled growth conditions. In *Plant, Cell & Environment* 41 (11), pp. 2549–2566. DOI: 10.1111/pce.13339.

**Bian, X.; Yao, L.; Si, E.; Meng, Y.; Li, B.; Ma, X. et al. 2022**: Characterization of glossy spike mutants and identification of candidate genes regulating cuticular wax synthesis in barley (*Hordeum vulgare* L.). In *International Journal of Molecular Sciences* 23 (21). DOI: 10.3390/ijms232113025.

**Bondada, B. R.; Oosterhuis, D. M.; Murphy, J. B.; Kim, K. S. 1996**: Effect of water stress on the epicuticular wax composition and ultrastructure of cotton (*Gossypium hirsutum* L.) leaf, bract, and boll. In *Environmental and Experimental Botany* 36 (1), pp. 61–69. DOI: 10.1016/0098-8472(96)00128-1.

**Bourdenx, B.; Bernard, A.; Domergue, F.; Pascal, S.; Léger, A.; Roby, D. et al. 2011**: Overexpression of Arabidopsis *ECERIFERUM1* promotes wax very-long-chain alkane biosynthesis and influences plant response to biotic and abiotic stresses. In *Plant Physiology* 156 (1), pp. 29–45. DOI: 10.1104/pp.111.172320.

**Bueno, A.; Alfarhan, A.; Arand, K.; Burghardt, M.; Deininger, A.-C.; Hedrich, R. et al. 2019b**: Effects of temperature on the cuticular transpiration barrier of two desert plants with water-spender and water-saver strategies. In *Journal of Experimental Botany* 70 (5), pp. 1613–1625. DOI: 10.1093/jxb/erz018.

**Bueno, A.; Sancho-Knapik, D.; Gil-Pelegrín, E.; Leide, J.; Peguero-Pina, J. J.; Burghardt, M.; Riederer, M. 2019a**: Cuticular wax coverage and its transpiration barrier properties in *Quercus coccifera* L. leaves: does the environment matter? In *Tree Physiology* 40 (7), pp. 827-840. DOI: 10.1093/treephys/tpz0110.

**Burghardt, M.; Burghardt, A.; Gall, J.; Rosenberger, C.; Riederer, M. 2008**: Ecophysiological adaptations of water relations of *Teucrium chamaedrys* L. to the hot and dry climate of xeric limestone sites in Franconia (Southern Germany). In *Flora - Morphology, Distribution, Functional Ecology of Plants* 203 (1), pp. 3–13. DOI: 10.1016/j.flora.2007.11.003.

**Burghardt, M.; Riederer, M. 2003**: Ecophysiological relevance of cuticular transpiration of deciduous and evergreen plants in relation to stomatal closure and leaf water potential. In *Journal of Experimental Botany* 54 (389), pp. 1941–1949. DOI: 10.1093/jxb/erg195.

**Cameron, K. D.; Teece, M. A.; Smart, L. B. 2006**: Increased accumulation of cuticular wax and expression of lipid transfer protein in response to periodic drying events in leaves of tree tobacco. In *Plant Physiology* 140 (1), pp. 176–183. DOI: 10.1104/pp.105.069724.

**Castellanos, I. C.; Rojas‐Pérez, L. C. 2024**: Extraction and characterisation of cuticular waxes from *Physalis peruviana* calyx. In *International Journal of Food Science and Technology* 59 (3), pp. 1829–1839. DOI: 10.1111/ijfs.16935.

**Chen, M.; Zhang, Y.; Kong, X.; Du, Z.; Zhou, H.; Yu, Z. et al. 2021**: Leaf cuticular transpiration barrier organization in tea tree under normal growth conditions. In *Frontiers in Plant Science* 12, p. 655799. DOI: 10.3389/fpls.2021.655799.

**Chen, M.; Zhu, X.; Zhang, Y.; Du, Z.; Chen, X.; Kong, X. et al. 2020**: Drought stress modify cuticle of tender tea leaf and mature leaf for transpiration barrier enhancement through common and distinct modes. In *Scientific Reports* 10 (1), p. 6696. DOI: 10.1038/s41598-020-63683-4.

**Chen, X.; Goodwin, S. M.; Boroff, V. L.; Liu, X.; Jenks, M. A. 2003**: Cloning and characterization of the WAX2 gene of Arabidopsis involved in cuticle membrane and wax production. In *The Plant Cell* 15 (5), pp. 1170–1185. DOI: 10.1105/tpc.010926.

**Cheng, G.; Ye, C.; Zhang, J.; Li, H.; Jiang, Y.; Duan, X. 2023**: Water permeability of different aerial tissues of carnations is related to cuticular wax composition. In *Physiologia Plantarum* 175 (2), e13883. DOI: 10.1111/ppl.13883.

**Darnell, R. L.; Ferree, D. C. 1983**: The influence of environment on apple tree growth, leaf wax formation, and foliar absorption. In *Journal of the American Society for Horticultural Science* 108 (3), pp. 506–511. DOI: 10.21273/JASHS.108.3.506.

**De Araújo Silva, M. M. de; Ferreira, L. T.; Vasconcelos, F. M. T. de; Willadino, L.; Camara, T. R.; dos Santos, D. Y. A. C.; Oliveira, A. F. M. de 2021**: Water stress-induced responses in the growth, cuticular wax composition, chloroplast pigments and soluble protein content, and redox metabolism of two genotypes of *Ricinus communis* L. In *Journal of Plant Growth Regulation* 40 (1), pp. 342–352. DOI: 10.1007/s00344-020-10103-6.

**DeLucia, E. H.; Berlyn, G. P. 1984**: The effect of increasing elevation on leaf cuticle thickness and cuticular transpiration in balsam fir. In *Canadian Journal of Botany* 62 (11), pp. 2423–2431. DOI: 10.1139/b84-331.

**Duursma, R. A.; Blackman, C. J.; Lopéz, R.; Martin-StPaul, N. K.; Cochard, H.; Medlyn, B. E. 2019**: On the minimum leaf conductance: its role in models of plant water use, and ecological and environmental controls. In *The New Phytologist* 221 (2), pp. 693–705. DOI: 10.1111/nph.15395.

**Elham, F.; Khavari-Nejad, A. R.; Salekdeh, G. H.; Farzaneh, N. 2012**: Evaluation of cuticular wax deposition, stomata and carbohydrate of wheat leaves for screening drought tolerance. In *Advances in Environmental Biology* 13 (6), pp. 4035–4040.

**Even, M.; Sabo, M.; Meng, D.; Kreszies, T.; Schreiber, L.; Fricke, W. 2018**: Night-time transpiration in barley (*Hordeum vulgare*) facilitates respiratory carbon dioxide release and is regulated during salt stress. In *Annals of Botany* 122 (4), pp. 569–582. DOI: 10.1093/aob/mcy084.

**Fricke, W.; Akhiyarova, G.; Wei, W.; Alexandersson, E.; Miller, A.; Kjellbom, P. O. et al. 2006**: The short-term growth response to salt of the developing barley leaf. In *Journal of Experimental Botany* 57 (5), pp. 1079–1095. DOI: 10.1093/jxb/erj095.

**Gauvrit, C.; Gaillardon, P. 1991**: Effect of low temperatures on 2,4‐D behaviour in maize plants. In *Weed Research* 31 (3), pp. 135–142. DOI: 10.1111/j.1365-3180.1991.tb01752.x.

**Giese, B. N. 1975**: Effects of light and temperature on the composition of epicuticular wax of barley leaves. In *Phytochemistry* 14 (4), pp. 921–929. DOI: 10.1016/0031-9422(75)85160-0.

**Go, Y. S.; Kim, H.; Kim, H. J.; Suh, M. C. 2014**: Arabidopsis Cuticular Wax Biosynthesis Is Negatively Regulated by the DEWAX Gene Encoding an AP2/ERF-Type Transcription Factor. In *The Plant Cell* 26 (4), pp. 1666–1680. DOI: 10.1105/tpc.114.123307.

**Gonzalez, R.; Paul, N. D.; Percy, K. E.; Ambrose, M.; McLaughlin, C. K.; Barnes, J. D. et al. 1996**: Responses to ultraviolet‐B radiation (280–315 nm) of pea (*Pisum sativum*) lines differing in leaf surface wax. In *Physiologia Plantarum* 98 (4), pp. 852–860. DOI: 10.1111/j.1399-3054.1996.tb06695.x.

**González, A.; Ayerbe, L. 2010**: Effect of terminal water stress on leaf epicuticular wax load, residual transpiration and grain yield in barley. In *Euphytica* 172 (3), pp. 341–349. DOI: 10.1007/s10681-009-0027-0.

**Grünhofer, P.; Herzig, L.; Schreiber, L. 2021**: Leaf morphology, wax composition, and residual (cuticular) transpiration of four poplar clones. In *Trees* 36 (2), pp. 645–658. DOI: 10.1007/s00468-021-02236-2.

**Grünhofer, P.; Herzig, L.; Sent, S.; Zeisler-Diehl, V. V.; Schreiber, L. 2022**: Increased cuticular wax deposition does not change residual foliar transpiration. In *Plant, Cell & Environment* 45 (4), pp. 1157–1171. DOI: 10.1111/pce.14274.

**Grünhofer, P.; Herzig, L.; Zhang, Q.; Vitt, S.; Stöcker, T.; Malkowsky, Y. et al. 2023**: Changes in wax composition but not amount enhance cuticular transpiration. In *Plant, Cell & Environment* 47 (1), pp. 91–105. DOI: 10.1111/pce.14719.

**Grünhofer, P.; Thangamani, P. D.; Schreiber, L.; Kreszies, T. 2025**: Sodium chloride enhances suberization in seminal roots but does not affect cutinized leaf barriers in cultivated and wild barley. In *Planta* 262 (2), p. 28. DOI: 10.1007/s00425-025-04743-9.

**Hadley, J. L.; William, S. K. 1990**: Influence of leaf surface wax and leaf area to water content ratio on cuticular transpiration in western conifers, U.S.A. In *Canadian Journal of Forest Research* 20 (9), pp. 1306–1311. DOI: 10.1139/x90-173.

**Hauke, V.; Schreiber, L. 1998**: Ontogenetic and seasonal development of wax composition and cuticular transpiration of ivy (*Hedera helix* L.) sun and shade leaves. In *Planta* 207, pp. 67–75. DOI: 10.1007/s004250050456.

**He, J.; Li, C.; Hu, N.; Zhu, Y.; He, Z.; Sun, Y. et al. 2022**: *ECERIFERUM1-6A* is required for the synthesis of cuticular wax alkanes and promotes drought tolerance in wheat. In *Plant Physiology* 190 (3), pp. 1640–1657. DOI: 10.1093/plphys/kiac394.

**He, J.; Tang, S.; Yang, D.;** **Chen, Y.; Ling, L.; Zou, Y. et al. 2019**: Chemical and transcriptomic analysis of cuticle lipids under cold stress in *Thellungiella salsuginea*. In *International Journal of Molecular Sciences* 20 (18). DOI: 10.3390/ijms20184519.

**Herzig, L.; Uellendahl, K.; Malkowsky, Y.; Schreiber, L.; Grünhofer, P. 2025**: In a different light: irradiation-induced cuticular wax accumulation fails to reduce cuticular transpiration. In *Plant, Cell & Environment* 20 (18), pp. 3632-3646. DOI: 10.1111/pce.15376.

**Holser, R. A. 2009**: Temperature‐dependent solubility of wax compounds in ethanol. In *European Journal of Lipid Science and Technology* 111 (10), pp. 1049–1052. DOI: 10.1002/ejlt.200900068.

**Huggins, T. D.; Mohammed, S.; Sengodon, P.; Ibrahim, A. M. H.; Tilley, M.; Hays, D. B. 2017**: Changes in leaf epicuticular wax load and its effect on leaf temperature and physiological traits in wheat cultivars (*Triticum aestivum* L.) exposed to high temperatures during anthesis. In *Journal of Agronomy and Crop Science* 204 (1), pp. 49–61. DOI: 10.1111/jac.12227.

**Hull, H. M. 1958**: The effect of day and night temperature on growth, foliar wax content, and cuticle development of velvet mesquite. In *Weeds* 6 (2), pp. 133–142. DOI: 10.2307/4040286.

**Hunsche, M.; Noga, G. 2011**: Cuticular wax load and surface wettability of leaves and fruits collected from sweet cherry (*Prunus avium*) trees grown under field conditions or inside a polytunnel. In *Acta Physiologiae Plantarum* 33 (5), pp. 1785–1792. DOI: 10.1007/s11738-011-0717-9.

**Islam, M. A.; Du, H.; Ning, J.; Ye, H.; Xiong, L. 2009**: Characterization of *Glossy1*-homologous genes in rice involved in leaf wax accumulation and drought resistance. In *Plant Molecular Biology* 70 (4), pp. 443–456. DOI: 10.1007/s11103-009-9483-0.

**Jefferson, P. G.; Johnson, D. A.; Rumbaugh, M. D.; Asay, K. H. 1989**: Water stress and genotypic effects on epicuticular wax production of Alfalfa and crested wheatgrass in relation to yield and excised leaf water loss rate. In *Canadian Journal of Plant Science* 69, pp. 481–490.

**Jenks, M. A.; Andersen, L.; Teusink, R. S.; Williams, M. H. 2001**: Leaf cuticular waxes of potted rose cultivars as affected by plant development, drought and paclobutrazol treatments. In *Physiologia Plantarum* 112 (1), pp. 62–70. DOI: 10.1034/j.1399-3054.2001.1120109.x.

**Jenks, M. A.; Joly, R. J.; Peters, P. J.; Rich, P. J.; Axtell, J. D.; Ashworth, E. N. 1994**: Chemically induced cuticle mutation affecting epidermal conductance to water vapor and disease susceptibility in *Sorghum bicolor* (L.) Moench. In *Plant Physiology* 105 (4), pp. 1239–1245. DOI: 10.1104/pp.105.4.1239.

**Jiang, J.; Zheng, X.; He, T.; Liu, X.; Zhao, Q.; Tan, W. et al. 2025**: The Function of PpKCS6 in Regulating Cuticular Wax Synthesis and Drought Resistance of Kentucky Bluegrass. In Plant, Cell & Environment 48 (6), pp. 4643–4655. DOI: 10.1111/pce.15465.

**Jordan, W. R.; Shouse, P. J.; Blum, A.; Miller, F. R.; Monk, R. L. 1984**: Environmental physiology of Sorghum. II. Epicuticular wax load and cuticular transpiration. In *Crop Science* 24 (6), pp. 1168–1173. DOI: 10.2135/cropsci1984.0011183X002400060038x.

**Kakani, V. G.; Reddy, K. R.; Zhao, D.; Mohammed, A. R. 2003**: Effects of ultraviolet-B radiation on cotton (*Gossypium hirsutum* L.) morphology and anatomy. In *Annals of Botany* 91 (7), pp. 817–826. DOI: 10.1093/aob/mcg086.

**Kerstiens, G. 1996**: Cuticular water permeability and its physiological significance. In *Journal of Experimental Botany* 47 (12), pp. 1813–1832. DOI: 10.1093/jxb/47.12.1813.

**Kim, H.; Go, Y. S.; Suh, M. C. 2018**: *DEWAX2* Transcription factor negatively regulates cuticular wax biosynthesis in Arabidopsis leaves. In *Plant & Cell Physiology* 59 (5), pp. 966–977. DOI: 10.1093/pcp/pcy033.

**Kim, K. S.; Park, S. H.; Jenks, M. A. 2007b**: Changes in leaf cuticular waxes of sesame (*Sesamum indicum* L.) plants exposed to water deficit. In *Journal of Plant Physiology* 164 (9), pp. 1134–1143. DOI: 10.1016/j.jplph.2006.07.004.

**Kim, K. S.; Park, S. H.; Kim, D. K.; Jenks, M. A. 2007a**: Influence of water deficit on leaf cuticular waxes of soybean (*Glycine max* [L.] Merr.). In *International Journal of Plant Sciences* 168 (3), pp. 307–316. DOI: 10.1086/510496.

**Koch, K.; Hartmann, K. D.; Schreiber, L.; Barthlott, W.; Neinhuis, C. 2006**: Influences of air humidity during the cultivation of plants on wax chemical composition, morphology and leaf surface wettability. In *Environmental and Experimental Botany* 56 (1), pp. 1–9. DOI: 10.1016/j.envexpbot.2004.09.013.

**Kosma, D. K.; Bourdenx, B.; Bernard, A.; Parsons, E. P.; Lü, S.; Joubès, J.; Jenks, M. A. 2009**: The impact of water deficiency on leaf cuticle lipids of Arabidopsis. In *Plant Physiology* 151 (4), pp. 1918–1929. DOI: 10.1104/pp.109.141911.

**Larsson, S.; Svenningsson, M. 1986**: Cuticular transpiration and epicuticular lipids of primary leaves of barley (*Hordeum vulgare*). In *Physiologia Plantarum* 68 (1), pp. 13–19. DOI: 10.1111/j.1399-3054.1986.tb06589.x.

**Le Provost, G.; Domergue, F.; Lalanne, C.; Campos, P. R.; Grosbois, A.; Bert, D. et al. 2013**: Soil water stress affects both cuticular wax content and cuticle-related gene expression in young saplings of maritime pine (*Pinus pinaster* Ait). In *BMC Plant Biology* 13 (95).

**Lee, S. B.; Kim, H.; Kim, R. J.; Suh, M. C. 2014**: Overexpression of Arabidopsis *MYB96* confers drought resistance in *Camelina sativa* via cuticular wax accumulation. In *Plant Cell Reports* 33 (9), pp. 1535–1546. DOI: 10.1007/s00299-014-1636-1.

**Leide, J.; Hildebrandt, U.; Reussing, K.; Riederer, M.; Vogg, G. 2007**: The developmental pattern of tomato fruit wax accumulation and its impact on cuticular transpiration barrier properties: effects of a deficiency in a beta-ketoacyl-coenzyme A synthase (*LeCER6*). In *Plant Physiology* 144 (3), pp. 1667–1679. DOI: 10.1104/pp.107.099481.

**Letchamo, W.; Gosselin, A. 1996**: Transpiration, essential oil glands, epicuticular wax and morphology of *Thymus vulgaris* are influenced by light intensity and water supply. In *Journal of Horticultural Science* 71 (1), pp. 123–134. DOI: 10.1080/14620316.1996.11515388.

**Lokesh, U.; Venkatesh, B.; Kiranmai, K.; Nareshkumar, A.; Amarnathareddy, V.; Rao, G. L. et al. 2019**: Overexpression of *ß-ketoacyl Co-A synthase1* gene improves tolerance of drought susceptible groundnut (*Arachis hypogaea* L.) cultivar K-6 by increased leaf epicuticular wax accumulation. In *Frontiers in Plant Science* 9, p. 1869. DOI: 10.3389/fpls.2018.01869.

**Luo, X.; Bai, X.; Sun, X.; Zhu, D.; Liu, B.; Ji, W. et al. 2013**: Expression of wild soybean *WRKY20* in Arabidopsis enhances drought tolerance and regulates ABA signalling. In *Journal of Experimental Botany* 64 (8), pp. 2155–2169. DOI: 10.1093/jxb/ert073.

**Luo, N.; Wang, Y.; Liu, Y.; Wang, Y.; Guo, Y.; Chen, C. et al. 2024**: 3-ketoacyl-CoA synthase 19 contributes to the biosynthesis of seed lipids and cuticular wax in Arabidopsis and abiotic stress tolerance. In Plant, Cell & Environment 47 (12), pp. 4599–4614. DOI: 10.1111/pce.15054.

**Lykholat, Y. V.; Khromykh, N. A.; Didur, O. O.; Alexeyeva, A. A.; Lykholat, T. Y. 2020**: Modification of the epicuticular waxes of plant leaves due to increased sunlight intensity. In *Biosys. divers.* 28 (1), pp. 29–33. DOI: 10.15421/012005.

**Mao, B.; Cheng, Z.; Lei, C.; Xu, F.; Gao, S.; Ren, Y. et al. 2012**: Wax crystal-sparse leaf2, a rice homologue of WAX2/GL1, is involved in synthesis of leaf cuticular wax. In *Planta* 235 (1), pp. 39–52. DOI: 10.1007/s00425-011-1481-1.

**Müller, Y.; Patwari, P.; Stöcker, T.; Zeisler-Diehl, V.; Steiner, U.; Campoli, C. et al. 2023**: Isolation and characterization of the gene *HvFAR1* encoding acyl-CoA reductase from the cer-za.227 mutant of barley (*Hordeum vulgare*) and analysis of the cuticular barrier functions. In *The New Phytologist* 239 (5), pp. 1903–1918. DOI: 10.1111/nph.19063.

**Ni, Y.; Guo, Y. J.; Han, L.; Tang, H.; Conyers, M. 2012**: Leaf cuticular waxes and physiological parameters in alfalfa leaves as influenced by drought. In *Photosynthetica* 50 (3), pp. 458–466. DOI: 10.1007/s11099-012-0055-1.

**Norris, R. F.; Bukovac, M. J. 1972**: Influence of cuticular waxes on penetration of pear leaf cuticle by 1‐naphthaleneacetic acid. In *Pesticide Science* 3 (6), pp. 705–708. DOI: 10.1002/ps.2780030607.

**Oliviera, A. F. M.; Meirelles, S. T.; Salatino, A. 2003**: Epicuticular waxes from caatinga and cerrado species and their efficiency against water loss. In *Anais da Academia Brasileira de Ciências* 75, pp. 431–439. DOI: 10.1590/S0001-37652003000400003.

**O'Toole, J. C.; Cruz, R. T.; Seiber, J. N. 1979**: Epicuticular Wax and Cuticular Resistance in Rice. In *Physiologia Plantarum* 47 (4), pp. 239–244. DOI: 10.1111/j.1399-3054.1979.tb06520.x.

**Patwari, P.; Salewski, V.; Gutbrod, K.; Kreszies, T.; Dresen-Scholz, B.; Peisker, H. et al. 2019**: Surface wax esters contribute to drought tolerance in Arabidopsis. In *The Plant Journal* 98 (4), pp. 727–744. DOI: 10.1111/tpj.14269.

**Pilon, J. J.; Lambers, H.; Baas, W.; Tosserams, M.; Rozema, J.; Atkin, O. K. 1999**: Leaf waxes of slow-growing alpine and fast-growing lowland *Poa* species: inherent differences and responses to UV-B radiation. In *Phytochemistry* 50 (4), pp. 571–580. DOI: 10.1016/S0031-9422(98)00556-1.

**Premachandra, G. S.; Saneoka, H.; Fujita, K.; Ogata, S. 1992**: Leaf water relations, osmotic adjustment, cell membrane stability, epicuticular wax load and growth as affected by increasing water deficits in Sorghum. In *Journal of Experimental Botany* 43 (12), pp. 1569–1576. DOI: 10.1093/jxb/43.12.1569.

**Qaderi, M. M.; Reid, D. M. 2005**: Growth and physiological responses of canola (*Brassica napus*) to UV‐B and CO2 under controlled environment conditions. In *Physiologia Plantarum* 125 (2), pp. 247–259. DOI: 10.1111/j.1399-3054.2005.00566.x.

**Qin, B. X.; Tang, D.; Huang, J.; Li, M.; Wu, X. R.; Lu, L. L. et al. 2011**: Rice *OsGL1-1* is involved in leaf cuticular wax and cuticle membrane. In *Molecular Plant* 4 (6), pp. 985–995. DOI: 10.1093/mp/ssr028.

**Rahman, T.; Shao, M.; Pahari, S.; Venglat, P.; Soolanayakanahally, R.; Qiu, X. et al. 2021**: Dissecting the roles of cuticular wax in plant resistance to shoot dehydration and low-temperature stress in Arabidopsis. In *International Journal of Molecular Sciences* 22 (4). DOI: 10.3390/ijms22041554.

**Reed, D. W.; Tukey, H. B. 1982**: Light intensity and temperature effects on epicuticular wax morphology and internal cuticle ultrastructure of carnation and brussels sprouts leaf cuticles. In *Journal of the American Society for Horticultural Science* 107 (3), pp. 417–420.

**Romero, P.; Rose, J. K. C. 2019**: A relationship between tomato fruit softening, cuticle properties and water availability. In *Food chemistry* 295, pp. 300–310. DOI: 10.1016/j.foodchem.2019.05.118.

**Sánchez, F. J.; Manzanares, M.; Andrés, E. F.; Tenorio, J. L.; Ayerbe, L. 2001**: Residual transpiration rate, epicuticular wax load and leaf colour of pea plants in drought conditions. Influence on harvest index and canopy temperature. In *European Journal of Agronomy* 15, pp. 57–70. DOI: 10.1016/S1161-0301(01)00094-6.

**Schlegel, T. K.; Schönherr, J.; Schreiber, L. 2005**: Size selectivity of aqueous pores in stomatous cuticles of *Vicia faba* leaves. In *Planta* 221 (5), pp. 648–655. DOI: 10.1007/s00425-005-1480-1.

**Schönherr, J.; Schreiber, L. 2004**: Size selectivity of aqueous pores in astomatous cuticular membranes isolated from *Populus canescens* (Aiton) Sm. leaves. In *Planta* 219 (3), pp. 405–411. DOI: 10.1007/s00425-004-1239-0.

**Schreiber, L.; Riederer, M. 1996**: Ecophysiology of cuticular transpiration: comparative investigation of cuticular water permeability of plant species from different habitats. In *Oecologia* 107, pp. 426–432. DOI: 10.1007/BF00333931.

**Schuster, A. C.; Burghardt, M.; Alfarhan, A.; Bueno, A.; Hedrich, R.; Leide, J. et al. 2016**: Effectiveness of cuticular transpiration barriers in a desert plant at controlling water loss at high temperatures. In *AoB PLANTS* 8. DOI: 10.1093/aobpla/plw027.

**Seo, P. J.; Lee, S. B.; Suh, M. C.; Park, M. J.; Go, Y. S.; Park, C. M. 2011**: The MYB96 transcription factor regulates cuticular wax biosynthesis under drought conditions in Arabidopsis. In *The Plant Cell* 23 (3), pp. 1138–1152. DOI: 10.1105/tpc.111.083485.

**Seo, P. J.; Park, C.-M. 2011**: Cuticular wax biosynthesis as a way of inducing drought resistance. In *Plant Signaling & Behavior* 6 (7), pp. 1043–1045. DOI: 10.4161/psb.6.7.15606.

**Shellakkutti, N.; Thangamani, P. D.; Suresh, K.; Baales, J.; Zeisler-Diehl, V.; Klaus, A. et al. 2022**: Cuticular transpiration is not affected by enhanced wax and cutin amounts in response to osmotic stress in barley. In *Physiologia Plantarum* 174 (4), e13735. DOI: 10.1111/ppl.13735.

**Simões, R.; Rodrigues, A.; Ferreira-Dias, S.; Miranda, I.; Pereira, H. 2020**: Chemical composition of cuticular waxes and pigments and morphology of leaves of *Quercus suber* trees of different provenance. In *Plants* 9 (9). DOI: 10.3390/plants9091165.

**Spoorthi, N.; Kavyashree, N. M.; Sushma, V.; Vijayaraghavareddy, P.; Sreeman, S. 2025**: Warm nights, waxy leaves: exploring the interplay between epicuticular wax and nocturnal transpiration. In *Rice Science*. DOI: 10.1016/j.rsci.2025.08.002.

**Steinmüller, D.; Tevini, M. 1985**: Action of ultraviolet radiation (UV-B) upon cuticular waxes in some crop plants. In *Planta* 164, pp. 557–564. DOI: 10.1007/BF00395975.

**Sumbur, B.; Zhou, M.; Dorjee, T.; Bing, J.; Ha, S.; Xu, X. et al. 2024**: Chemical and transcriptomic analyses of leaf cuticular wax metabolism in *Ammopiptanthus mongolicus* under Osmotic Stress. In *Biomolecules* 14 (2). DOI: 10.3390/biom14020227.

**Sutter, E.; Langhans, R. W. 1982**: Formation of epicuticular wax and its effect on water loss in cabbage plants regenerated from shoot-tip culture. In *Canadian Journal of Botany* 60 (12), pp. 2896–2902. DOI: 10.1139/b82-350.

**VanderWeide, J.; Yan, Y.; Zandberg, W. F.; Castellarin, S. D. 2022**: Modulation of grape cuticular wax composition following multiple heatwaves influences grape transpiration. In *Environmental and Experimental Botany* 202, 105036. DOI: 10.1016/j.envexpbot.2022.105036.

**Vogg, G.; Fischer, S.; Leide, J.; Emmanuel, E.; Jetter, R.; Levy, A. A.; Riederer, M. 2004**: Tomato fruit cuticular waxes and their effects on transpiration barrier properties: functional characterization of a mutant deficient in a very-long-chain fatty acid beta-ketoacyl-CoA synthase. In *Journal of Experimental Botany* 55 (401), pp. 1401–1410. DOI: 10.1093/jxb/erh149.

**Wang, W.; Zhang, Y.; Xu, C.; Ren, J.; Liu, X.; Black, K. et al. 2015a**: Cucumber *ECERIFERUM1* (*CsCER1*), which influences the cuticle properties and drought tolerance of cucumber, plays a key role in VLC alkanes biosynthesis. In *Plant Molecular Biology* 87 (3), pp. 219–233. DOI: 10.1007/s11103-014-0271-0.

**Wang, W.; Liu, X.; Gai, X.; Ren, J.; Liu, X.; Cai, Y. et al. 2015b**: *Cucumis sativus* L. *WAX2* Plays a pivotal role in wax biosynthesis, influencing pollen fertility and plant biotic and abiotic stress responses. In *Plant & Cell Physiology* 56 (7), pp. 1339–1354. DOI: 10.1093/pcp/pcv052.

**Wang, Y.; Sun, Y.; You, Q.; Luo, W.; Wang, C.; Zhao, S. et al. 2018**: Three Fatty Acyl-Coenzyme A Reductases, BdFAR1, BdFAR2 and BdFAR3, are Involved in Cuticular Wax Primary Alcohol Biosynthesis in *Brachypodium distachyon*. In *Plant & Cell Physiology* 59 (3), pp. 527–543. DOI: 10.1093/pcp/pcx211.

**Wang, Y.; Wan, L.; Zhang, L.; Zhang, Z.; Zhang, H.; Quan, R. et al. 2012**: An ethylene response factor OsWR1 responsive to drought stress transcriptionally activates wax synthesis related genes and increases wax production in rice. In *Plant Molecular Biology* 78 (3), pp. 275–288. DOI: 10.1007/s11103-011-9861-2.

**Whitecross, M. I.; Armstrong, D. J. 1972**: Environmental effects on epicuticular waxes of *Brassica napus* L. In *Australian Journal of Botany* 20 (1), pp. 87–95. DOI: 10.1071/BT9720087.

**Xu, H.; Liu, X.; Ban, Z.; Yang, Y.; Zhang, L.; Bi, Q.; Wang, L. 2025**: XsLTPG31 confers leaf cuticular wax deposition and drought resistance in Yellowhorn. In *Plant, Cell & Environment* 48 (12), pp. 8518–8533. DOI: 10.1111/pce.70144.

**Yang, F.; Han, Y.; Zhu, Q.-H.; Zhang, X.; Xue, F.; Li, Y. et al. 2022**: Impact of water deficiency on leaf cuticle lipids and gene expression networks in cotton (*Gossypium hirsutum* L.). In *BMC Plant Biology* 22 (1), p. 404. DOI: 10.1186/s12870-022-03788-2.

**Yang, J.; Isabel Ordiz, M.; Jaworski, J. G.; Beachy, R. N. 2011a**: Induced accumulation of cuticular waxes enhances drought tolerance in Arabidopsis by changes in development of stomata. In *Plant physiology and Biochemistry* 49 (12), pp. 1448–1455. DOI: 10.1016/j.plaphy.2011.09.006.

**Yang, M.; Yang, Q.; Fu, T.; Zhou, Y. 2011b**: Overexpression of the *Brassica napus BnLAS* gene in Arabidopsis affects plant development and increases drought tolerance. In *Plant Cell Reports* 30 (3), pp. 373–388. DOI: 10.1007/s00299-010-0940-7.

**Zeisler-Diehl, V. V.; Migdal, B.; Schreiber, L. 2017**: Quantitative characterization of cuticular barrier properties: methods, requirements, and problems. In *Journal of Experimental Botany* 68 (19), pp. 5281–5291. DOI: 10.1093/jxb/erx282.

**Zhang, J. Y.; Broeckling, C. D.; Blancaflor, E. B.; Sledge, M. K.; Sumner, L. W.; Wang, Z. Y. 2005**: Overexpression of *WXP1*, a putative *Medicago truncatula* AP2 domain-containing transcription factor gene, increases cuticular wax accumulation and enhances drought tolerance in transgenic alfalfa (*Medicago sativa*). In *The Plant Journal* 42 (5), pp. 689–707. DOI: 10.1111/j.1365-313X.2005.02405.x.

**Zhang, J. Y.; Broeckling, C. D.; Sumner, L. W.; Wang, Z. Y. 2007**: Heterologous expression of two *Medicago truncatula* putative ERF transcription factor genes, *WXP1* and *WXP2*, in Arabidopsis led to increased leaf wax accumulation and improved drought tolerance, but differential response in freezing tolerance. In *Plant Molecular Biology* 64 (3), pp. 265–278. DOI: 10.1007/s11103-007-9150-2.

**Zhang, P.; Zou, Y.; Song, B.; Zhou, M.; He, J.; Chen, Y. et al. 2022**: Cuticular lipids and associated gene expression analysis under NaCl stress in *Thellungiella salsuginea*. In *Physiologia Plantarum* 174 (1), e13625. DOI: 10.1111/ppl.13625.

**Zhang, Y.; Du, Z.; Han, Y.; Chen, X.; Kong, X.; Sun, W. et al. 2021**: Plasticity of the cuticular transpiration barrier in response to water shortage and resupply in *Camellia sinensis*: A Role of Cuticular Waxes. In *Frontiers in Plant Science* 11, p. 600069. DOI: 10.3389/fpls.2020.600069.

**Zhang, Y. L.; Zhang, C. L.; Wang, G. L.; Wang, Y. X.; Qi, C. H.; You, C. X. et al. 2019**: Apple AP2/EREBP transcription factor MdSHINE2 confers drought resistance by regulating wax biosynthesis. In *Planta* 249 (5), pp. 1627–1643. DOI: 10.1007/s00425-019-03115-4.

**Zhao, Q. P.; Miao, B. L.; Zhu, J. D.; Li, X. K.; Fu, X. L.; Han, M. Y. et al. 2025**: Sec24C Participates in Cuticular Wax Transport by Facilitating Plasma Membrane Localization of ABCG5. In Plant, Cell & Environment 48 (5), pp. 3012–3026. DOI: 10.1111/pce.15320.

**Zhou, M.; Li, D.; Li, Z.; Hu, Q.; Yang, C.; Zhu, L.; Luo, H. 2013a**: Constitutive expression of a *miR319* gene alters plant development and enhances salt and drought tolerance in transgenic creeping bentgrass. In *Plant Physiology* 161 (3), pp. 1375–1391. DOI: 10.1104/pp.112.208702.

**Zhou, L.; Ni, E.; Yang, J.; Zhou, H.; Liang, H.; Li, J. et al. 2013b**: Rice *OsGL1-6* is involved in leaf cuticular wax accumulation and drought resistance. In *PloS one* 8 (5), e65139. DOI: 10.1371/journal.pone.0065139.

**Zhu, L.; Guo, J.; Zhu, J.; Zhou, C. 2014**: Enhanced expression of EsWAX1 improves drought tolerance with increased accumulation of cuticular wax and ascorbic acid in transgenic Arabidopsis. In *Plant Physiology and Biochemistry* 75, pp. 24–35. DOI: 10.1016/j.plaphy.2013.11.028.
